# A preliminary phylogeny and review of the genus *Tasmanitachoides*, with descriptions of two new species (Coleoptera, Carabidae, Bembidarenini)

**DOI:** 10.3897/zookeys.1044.62253

**Published:** 2021-06-16

**Authors:** David R. Maddison, Nick Porch

**Affiliations:** 1 Department of Integrative Biology, Oregon State University, Corvallis, OR 97331, USA Oregon State University Corvallis United States of America; 2 School of Life and Environmental Sciences, Deakin University, Geelong 3216, Australia Deakin University Geelong Australia

**Keywords:** Australia, beetle, DNA, systematics, taxonomy, Trechinae

## Abstract

The genus *Tasmanitachoides* Erwin, a genus of very small carabid beetle endemic to Australia, is reviewed. Although uncommon in collections, they can be abundant and diverse on banks of fine gravel or coarse sand next to bodies of fresh water; samples from southeastern Australia suggest numerous undescribed species. An initial phylogenetic hypothesis for the genus is presented, including 19 of the 32 known species. The inferred phylogeny, based upon one mitochondrial and four nuclear genes, shows the *kingi* group to be sister to remaining *Tasmanitachoides*, with the *wattsensis* group and *T. lutus* (Darlington) also being phylogenetically isolated. Two new species are described: *T. baehri***sp. nov.**, from the Australian Capital Territory, is a member of the *kingi* group; *T. erwini***sp. nov.**, from Tasmania, is a member of the *wattsensis* group. Identification tools for described and some undescribed species are presented, including photographs of all known species.

## Introduction

**Figure 1.**
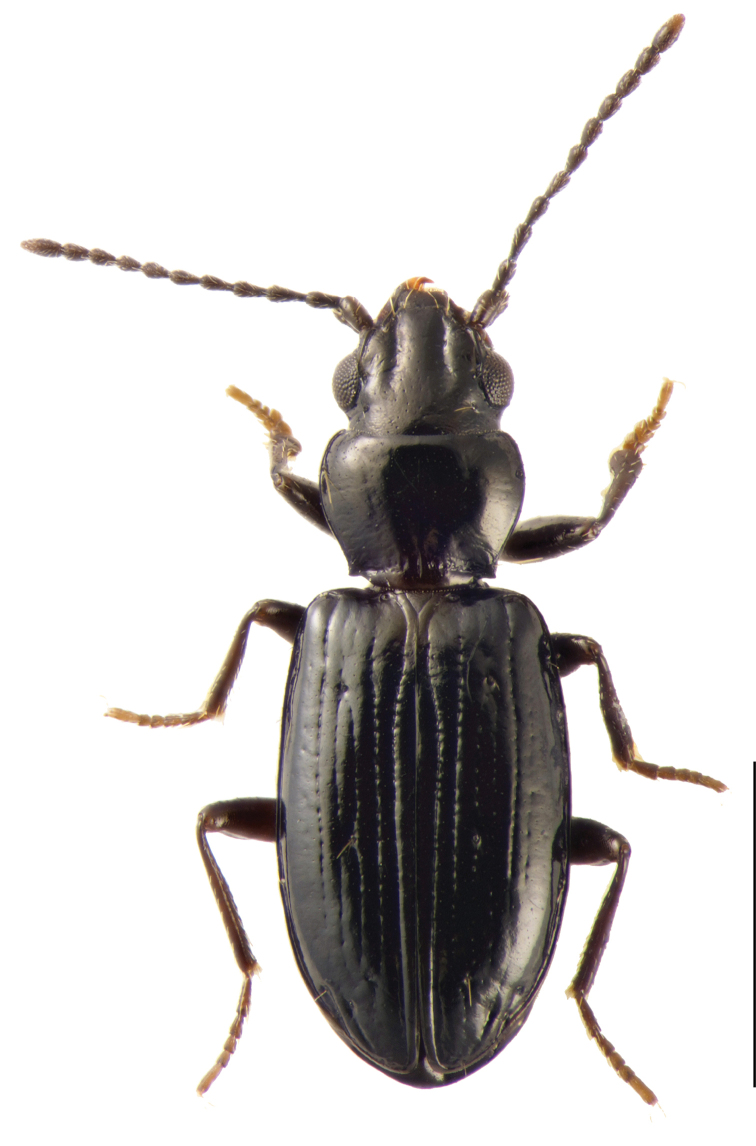
*Tasmanitachoides erwini* sp. nov., adult male, from the type locality. Scale bar: 1 mm.

The genus *Tasmanitachoides* Erwin, 1972 (Fig. 1) comprises very small carabid beetles found on fine gravel and coarse sand shores of bodies of fresh water throughout Australia. *Tasmanitachoides* was proposed to include six species described between 1895 and 1962 by Thomas G. Sloane (1895, 1896, 1921), Thomas Blackburn (1901), and Philip J. Darlington (1962), plus two additional species described by Erwin (1972). Martin Baehr (1990) revised the genus, adding five species, followed by a later series of papers (Baehr 2001, 2008a, b, 2009, 2010, 2013) in which he brought the total of known species to 25.

This enigmatic group has migrated through the classification of carabids. In describing the first known species, which he called *Tachys murrumbidgensis*, Sloane (1895) noted “I am in some doubt as to the position of this species… It seems a thoroughly isolated species”; Blackburn (1901), in contrast, considered the two species he described to belong to *Bembidion*. Darlington (1962) noted that “Although they are certainly *Tachys* rather than *Bembidion* by current classification, the species of this group are anomalous (primitive ?) in some ways and should be specially considered by students of bembidiine phylogeny”. Erwin (1972) recognized that they were not *Tachys*, but instead considered them “an early off-shoot of the tachyine lineage which gave rise to the Anillina.” However, DNA sequences and morphological data (Grebennikov 2008; Maddison and Ober 2011; Maddison et al. 2019) convincingly indicate that they are not closely related to either *Bembidion* or tachyines, but instead are members of a Southern Hemisphere clade including the South American genera *Bembidarenas*, *Argentinatachoides*, and *Andinodontis*; this clade of four genera is now known as the isolated tribe Bembidarenini (Maddison et al. 2019).

During the last twenty years, two threads wove together to yield the discovery, documented here, that *Tasmanitachoides* are likely notably more diverse than reported in the 13 papers describing the 25 known species. NP began collecting *Tasmanitachoides* in 1998, intrigued by their described diversity given the relatively few specimens that had been studied by Martin Baehr. Baehr (1990) had documented 16 species based upon an examination of only 157 specimens; nine of the species were known from only 1–6 specimens each. These limited numbers suggest either a rarity in nature, a cryptic habitat, a lack of collecting effort, or a combination of these factors. Both this paucity of studied specimens and the difficulty NP encountered in identifying his specimens hinted at a potential for undocumented diversity. Separately, the first DNA sequence data of *Tasmanitachoides fitzroyi* Darlington acquired by DRM in 2000 showed that it was not a tachyine, and that it held an isolated position within the supertribe Trechitae. A desire to confirm and extend the DNA results led to a collaboration between us, with NP collecting a small series of specimens of *Tasmanitachoides* from the Murrumbidgee River at Uriarra Crossing in 2002. The diversity of species revealed by the DNA sequence data and associated morphological study suggested that in that small sample there was at least one undescribed species, and possibly a second. We posited that this genus held hidden diversity, perhaps unnoticed because the beetles’ very small size made both their capture and their examination difficult. A plan was formed to more seriously re-examine the diversity of *Tasmanitachoides* using both DNA sequence data and morphological structures, but it was not until 2019, when DRM visited NP in Australia, that the project gained momentum.

After two decades of pondering *Tasmanitachoides*, our first encounter together with living *Tasmanitachoides* was memorable. DRM had collected numerous species of the three South American genera of Bembidarenini on trips to Chile, Argentina, and Ecuador between 2006 and 2011, but had only seen preserved *Tasmanitachoides*. As neontological systematists we often are embedded in rooms full of dead carcasses of the organisms we study, and as beautiful as they may be, the experience of observing pinned specimens is very different than seeing alive, in nature, the biodiversity we seek to document and discover. In the early evening of 8 January 2019, we approached Uriarra Crossing of the Murrumbidgee River, not knowing whether we would find these relatively rarely collected organisms. At our first footsteps on the fine gravel banks of the river specimens of *Tasmanitachoides* emerged from the substrate, and in short order we had collected three species and more than 110 individuals.

**Figure 2.**
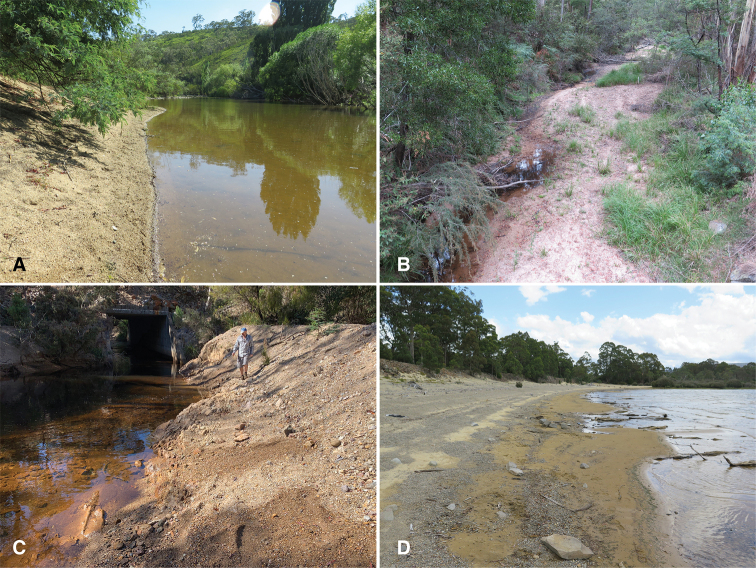
Habitats of *Tasmanitachoides***A** Australia: ACT: Murrumbidgee River at Angle Crossing, 35.5825°S, 149.1100°E, 598 m. This and similar areas a few meters upstream are habitat of *T. murrumbidgensis*, *T.* sp. “ Tambo R”, T. cf. gerdi, *T.* sp. “Angle Crossing #1”, *T. wilsoni*, *T. maior*, *T.* sp. “Angle Crossing #2”, and *T. rufescens***B** Australia: Victoria: Flat Rock Creek at highway B23, 37.2835°S, 149.2223°E, 256 m. Habitat of *T. lutus*, *T. leai*, and *T. angulicollis***C** Australia: Tasmania: mouth of Machinery Creek into the River Forth at C136, 41.4712°S, 146.1366°E, 126 m. Habitat of *T. leai*, *T. kingi*, *T. erwini*, and *T.* sp. “River Forth” **D** Australia: Tasmania: Lake St Clair, 42.1121°S, 146.2051°E, 741 m. Habitat of *T. hobarti*.

These beetles are not rare in their preferred habitat (Fig. 2). We found them to be common at the ten sites we visited (two on the Murrumbidgee River in the Australian Capital Territory, three in Victoria, and five in Tasmania). Almost all collecting was during daylight hours. At some localities *Tasmanitachoides* were so abundant that the limiting factor was not finding specimens but capturing them; with dozens of specimens per square meter, many would escape as we were collecting other specimens. In total, we found more than 1100 specimens at those ten sites, representing 15 species, at least four of which are undescribed. It became evident that the group was poorly collected. For example, in Tasmania, we found *T. leai* (Sloane) to be abundant at three of our five sites, but it had yet to be reported from the island in the literature; although we found more than 200 specimens of *T. hobarti* (Blackburn) at four of our sites in Tasmania, these apparently were the first specimens collected since the type series at least 118 years earlier.

*Tasmanitachoides* are found on the shores of larger rivers (Fig. 2A; see also the 360° view at https://goo.gl/maps/gCpfBCHxueUCr3Kf9), or smaller, more shaded upland creeks (Fig. 2B), or smaller rivers (Fig. 2C), or lake shores (Fig. 2D), with different species appearing to prefer different elevations, levels of shade, water flow regimes, and water body sizes. At Angle Crossing on the Murrumbidgee River in the Australian Capital Territory (Fig. 2A), we found eight species, but at the smaller and more shaded Flat Rock Creek in eastern Victoria (Fig. 2B) we found a different fauna, with three other species. In Tasmania, Lake St. Clair had only *Tasmanitachoides hobarti* on its banks, whereas the mouth of Machinery Creek into the River Forth (Fig. 2C) had four other species, but no *T. hobarti*. There are also differences in species distributions among microhabitats at a single site. For example, at the Machinery Creek / River Forth site (Fig. 2C), *T. kingi* Darlington was found in drier areas higher up on the bank, approximately 1.5–2 m from the water, whereas *T. leai* was found primarily lower and closer to the water.

**Figure 3.**
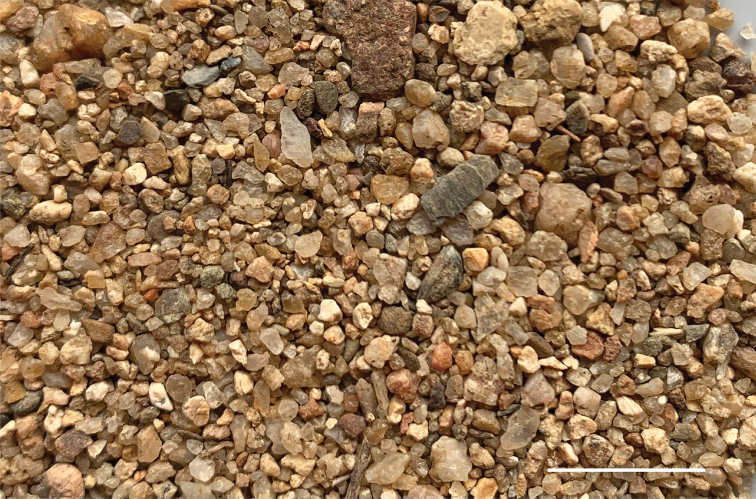
Gravel from *Tasmanitachoides* habitat at Australia: ACT: Murrumbidgee River at Angle Crossing, 35.5803°S, 149.1109°E, 600 m. This is the substrate from an area in which *T. murrumbidgensis* and *T.* sp. “Tambo R” were abundant, with some specimens of *T. maior* and *T. rufescens*. Scale bar: 10 mm.

**Figure 4.**
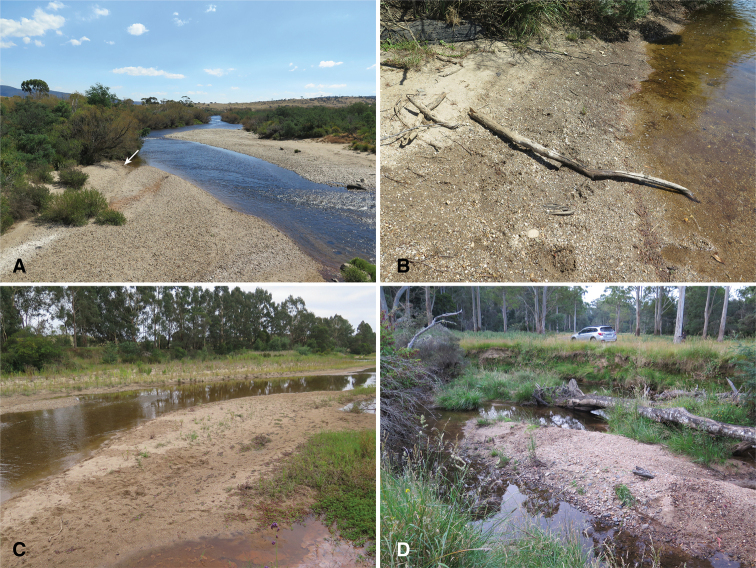
Habitats of *Tasmanitachoides***A, B** Australia: Tasmania: South Esk River at Avoca, 41.7807°S, 147.7148°E, 193 m. Habitat of *T. leai*, *T. hobarti*, and *T.* sp. “River Forth”. We found *Tasmanitachoides* only in a small area noted by the arrow in **A**, and shown close-up in **B C** Australia: Victoria: Tambo River at Bruthen, 37.7111°S, 147.8369°E, 10 m. Habitat of *T. murrumbidgensis*, *T.* sp. “Tambo R”, *T. maior*, as well as *Bembidion aterdustum*, *B. brullei*, *B. jacksoniense*, and *Tachyura victoriensis***D** Australia: Tasmania: Weld River NE Weldborough, 41.1897°S, 147.9118°E, 357 m. Habitat of *T. hobarti*.

In these water-shore habitats, *Tasmanitachoides* are concentrated in those regions with no or minimal vegetation, within 3 meters of the shoreline, with at least some moisture a centimeter or two below the surface. Most critically, though, they are found where the substrate is composed of moderately well-sorted fine gravel and coarse sand, with particles mostly approximately 1–4 mm (Fig. 3), with at most small amounts of finer sand, silt, or clay mixed in. This substrate is extremely porous, such that water splashed upon it quickly drains through. The beetles emerge when water is poured on the surface; they then run up the bank. Microhabitats with clean, fine gravel can be widespread at a site, or quite localized. For example, along the South Esk River at Avoca we found *Tasmanitachoides* in only one small patch a few meters long (Fig. 4A, B). This patch was composed of clean, fine gravel, and had many *Tasmanitachoides* (we collected more than 60). In other rivers and creeks, they were extensively distributed along much of the gravel shoreline (e.g., Figs 2, 4C, D).

This is the first in a series of planned papers about diversity within *Tasmanitachoides*. We infer an initial phylogeny of the genus based upon DNA sequence data, document some aspects of the diversity we found, describe two new species, and provide an improved identification key as well as images of the species. We plan a more complete revision of the genus after more focused collecting throughout Australia, and a more detailed phylogenetic analysis including more species.

## Materials and methods

Members of *Tasmanitachoides* were examined from or deposited in the collections listed below. Each collection’s listing begins with the code used in the text.

**ANIC**Australian National Insect Collection, Canberra, Australia;

**MNHN**Muséum National d’Histoire Naturelle, Paris, France;

**MCZ**Museum of Comparative Zoology, Harvard University, Cambridge, USA;

**NHMUK**The Natural History Museum, London, UK;

**NMV**Museums Victoria, Melbourne, Australia;

**NPC** Nick Porch Collection, Melbourne, Australia;

**OSAC**Oregon State Arthropod Collection, Oregon State University, Corvallis, USA;

**QVMAG**Queen Victoria Museum and Art Gallery, Launceston, Australia;

**TMAG**Tasmanian Museum and Art Gallery;

**USNM**National Museum of Natural History, Smithsonian Institution, Washington, DC, USA;

**ZSM**Zoologische Staatssammlung München, Munich, Germany.

### Collecting methods

Specimens were collected with the aid of an aspirator after splashing water on fine gravel or coarse sand, and waiting for the beetles to appear on the surface. Specimens for morphological studies were killed and preserved in *Acer* sawdust to which ethyl acetate was added. Specimens for DNA sequencing were collected into 95% or 100% ethanol.

### Morphological methods

General methods of specimen preparation for morphological work, and terms used, follow Maddison (1993; 2008). Genitalia were prepared, after dissection from the body, by treatment in 10% KOH at 65 °C for 10 minutes followed by multi-hour baths of distilled water, 5% glacial acetic acid, distilled water, and then ethanol. Male genitalia were then mounted in Euparal on a small circular coverslip attached to archival-quality heavyweight watercolor paper, and, once dried, pinned beneath the specimen.

Photographs of entire beetles, elytra, and heads were taken with a Leica M165C dissecting scope and a Sony NEX-7 camera, and of male genitalia with a Leica DM5500B compound microscope and DMC425C camera. Microsculpture photographs were taken with a DMC425C camera attached to a DM5500B compound scope equipped with an X-Cite 110LED light source, which provides co-axial illumination, and a 20× epi-illumination objective lens. For all photographs of specimens or body parts, a stack of images from different focal positions was merged using the PMax procedure in Zerene Systems’ Zerene Stacker; the final images thus potentially have some artefacts caused by the merging algorithm. Measurements were made using Leica Application Suite v4.9 from images acquired using these either a Leica Z6 Apo lens and DMC4500 camera or a Leica DM5500B compound microscope and DMC425C camera.

We follow Baehr (1990) in measuring the body length of specimens from the anterior edge of the labrum to the tip of the longest elytron.

### Molecular methods

Genes studied, and abbreviations used in this paper, are:

**28S** 28S ribosomal DNA (D1–D3 domains);

**18S** 18S ribosomal DNA;

**CAD4** and **CAD2** carbamoyl phosphate synthetase domain of the *rudimentary* gene (part 4 and part 2 of Moulton and Wiegmann 2004);

**COI** cytochrome c oxidase I;

**wg** wingless.

DNA was extracted using a Qiagen DNeasy Blood and Tissue Kit. Gene fragments were amplified using the Polymerase Chain Reaction on an Eppendorf Mastercycler ProS Thermal Cycler, using TaKaRa Ex Taq and the basic protocols recommended by the manufacturers. Primers and details of the cycling reactions used are given in Maddison et al. (2019), with the addition that some of the 18S sequences were amplified with primers 18S5 (GACAACCTGGTTGATCCTGCCAGT) and 18Sb5 (TAACCGCAACAACTTTAAT) (Shull et al. 2001) using two rounds of cycling, with the first round beginning with an annealing temperature of 51 °C, which was sequentially reduced by 0.5 °C for each of 10 cycles, and the second round of 27 cycles using an annealing temperature of 46 °C; all cycles used an extension time of 60 seconds. The amplified products were then cleaned, quantified, and sequenced at the University of Arizona’s Genomic and Technology Core Facility using a 3730 XL Applied Biosystems automatic sequencer. Assembly of multiple chromatograms for each gene fragment and initial base calls were made with Phred (Green and Ewing 2002) and Phrap (Green 1999) as orchestrated by Mesquite’s Chromaseq package (Maddison and Maddison 2020a, c) with subsequent modifications by Chromaseq and manual inspection. Multiple peaks at a single position in multiple reads were coded using IUPAC ambiguity codes.

We sampled DNA from 54 specimens of 19 species of *Tasmanitachoides*, as well as specimens of seven outgroup species, which belonged to other genera of Bembidarenini (Table 1). Of the 214 sequences examined, 153 were newly acquired, with 61 being from previous publications (Maddison and Ober 2011; Maddison 2012; Kanda et al. 2015; Maddison, et al. 2019). Of the 19 species of *Tasmanitachoides* sampled, we consider seven to belong to undescribed species (*T. erwini* sp. nov., *T. baehri* sp. nov., *T.* sp. “Lerderderg R”, *T.* sp. “Angel Crossing #1”, *T.* sp. “Angel Crossing #2”, *T.* sp. “River Forth”, and *T.* sp. “Tambo R”), the first two of which are described in this paper. Locality information for the *Tasmanitachodes* whose DNA was sequenced is provided in Table 2. For *Tasmanitachoides fitzroyi*, the terminal taxon used in the analyses is a chimera of two different specimens from the same locality, with 28S from specimen DNA0762 and the remaining genes from DNA1575. Sequences of the two holotypes listed in Table 1 are “genseq-1”, of paratypes “genseq-2”, and the remainder are all “genseq-4” (Chakrabarty et al. 2013).

**Table 1.** Specimens and genes sequenced of Bembidarenini. Four-digit numbers in entries (#) are D.R. Maddison DNA voucher numbers; further information about *Tasmanitachoides* specimens is given in Table 2. Other entries are GenBank accession numbers. DNA5569 under *T. baehri* sp. nov. and DNA5509 under *T. erwini* sp. nov. are holotypes; the remaining specimens listed for those two species are paratypes.

	#	28S	COI	wg	CAD4	CAD2	18S
*** Bembidarenas ***							
*Bembidarenas reicheellum*	1450	KU233745	KU233799	KU233874	KU233912	MK118232	KU233699
Bembidarenas sp. nr. reicheellum	2213	JN170274	JN170980	JN171345	JN170740	MK118277	JN170140
*** Argentinatachoides ***							
*Argentinatachoides balli*	2279	MK103971	MW291247	MK118571	MK112132	MK118280	MK103912
*Argentinatachoides setiventre*	2214		JN170981	JN171346	JN170741		JN170141
	2226	JN170275				MK118278	
*Argentinatachoides* sp. “Argentina: Neuquen”	2326	MK103973		MK118573	MK112135	MK118286	MK103913
*** Andinodontis ***							
*Andinodontis muellermotzfeldi*	2654	MK103993	MW291248	MK118592	MK112154	MK118303	MK103918
*Andinodontis* sp. “Ecuador: Vinillos”	2665	MK103992	MW291249	MK118593	MK112153	MK118306	MK103917
*** Tasmanitachoides ***							
*T. kingi* (Darlington)	5489	MW291161	MW291255	MW291210	MW291230		MW291301
	5753	MW291162	MW291256				
*T. angulicollis* Baehr	5515	MW291163	MW291257	MW291211	MW291231		MW291302
*T. wilsoni* (Sloane)	5514	MW291164	MW291258				
	5580	MW291165	MW291259	MW291212	MW291232		MW291303
*T. baehri* sp. nov.	2032	MK103966	MW291250	MK118567	MK112126	MK118265	MK103911
	5569	MW291166	MW291260	MW291213			MW291304
*T.* sp. “Lerderderg R”	1772	MK103950	MW291253	MK118551	MK112108	MK118250	MW291299
	2029	MW291168	MW291261	MW291214	MW291233	MW291296	
	5584	MW291169					
*T. erwini* sp. nov.	5509	MW291170	MW291262	MW291215	MW291234		MW291305
	5583	MW291171	MW291263	MW291216	MW291235		
	5679	MW291172	MW291264	MW291217	MW291236		
*T. lutus* (Darlington)	5582	MW291167					
	1773	MK103951	MW291251	MK118552	MK112109	MK118251	MK103907
T. cf. gerdi Baehr	5575	MW291173	MW291265				
	2030	MW291174	MW291266	MW291218	MW291237	MW291297	MW291306
	5556	MW291175					
	5676	MW291176	MW291267				
*T.* sp. “Angle Crossing #1”	5497	MW291177					
	5578	MW291178	MW291268	MW291219	MW291238		MW291307
	5677	MW291179	MW291269				
*T. hobarti* (Blackburn)	5551	MW291180					
	5554	MW291181					
	5579	MW291182					
	5488	MW291183	MW291270	MW291220	MW291239		
	5552	MW291184					
*T. leai* (Sloane)	5507	MW291185	MW291271				
	5525	MW291186	MW291272				
	5549	MW291187	MW291273				
	5557	MW291188	MW291274	MW291221	MW291240		
	5581	MW291189	MW291275	MW291222	MW291241		MW291308
*T. rufescens* Baehr	1993	MW291190	MW291276	MW291223	MW291242	MW291298	MW291309
	5577	MW291191	MW291277				
*T.* sp. “River Forth”	5555	MW291192	MW291278	MW291224	MW291243		MW291310
*T.* sp. “Angle Crossing #2”	5550	MW291193	MW291279	MW291225	MW291244		MW291311
	5678	MW291194	MW291280				
*T. bicolor* Baehr	5568	MW291195	MW291281				
*T. fitzroyi* (Darlington)	0762	GU556122					
	1575		MW291252	MK118542	MK112097	MK118237	GU556148
*T. maior* Baehr	5508	MW291196	MW291282	MW291226	MW291245		
	5567	MW291197	MW291283	MW291227			MW291312
*T. murrumbidgensis* (Sloane)	2031	MK103965	MW291254	MK118566	MK112125	MK118264	MW291300
	5553	MW291198	MW291284				
	5564	MW291199	MW291285				
	5565	MW291200	MW291286	MW291228			
	5566	MW291201	MW291287				
	5571	MW291202	MW291288				
	5572	MW291203	MW291289				
	5754	MW291204	MW291290				
*T.* sp. “Tambo R”	5548	MW291205	MW291291	MW291229	MW291246		MW291313
	5570	MW291206	MW291292				
	5573	MW291207	MW291293				
	5574	MW291208	MW291294				
	5576	MW291209	MW291295				

Alignment was not difficult for any of the protein-coding genes. There were no insertion or deletions (indels) evident in the sampled CAD4, CAD2, wg, or COI sequences. Alignments of 28S and 18S were conducted in MAFFT version 7.130b (Katoh and Standley 2013), using the L-INS-i search option and otherwise default parameter values.

**Table 2.** Localities of capture of *Tasmanitachoides* specimens whose DNA was sequenced. Four-digit numbers at the start of each row are D.R. Maddison DNA voucher numbers.

***Tasmanitachoides kingi* (Darlington)**	
5489	Australia: TAS: River Forth at C136, 126 m, 41.4712°S, 146.1366°E
5753	Australia: TAS: Weld River NE Weldborough, 357 m, 41.1897°S, 147.9109°E
***Tasmanitachoides angulicollis* Baehr**	
5515	Australia: VIC: Flat Rock Creek at highway B23, 256 m, 37.2835°S, 149.2223°E
***Tasmanitachoides wilsoni* (Sloane)**	
5514	Australia: ACT: Murrumbidgee R. at Uriarra Crossing, 450 m, 35.2462°S, 148.9530°E
5580	Australia: ACT: Murrumbidgee R. at Angle Crossing, 598 m, 35.5825°S, 149.1100°E
***Tasmanitachoides baehri* sp. nov.**	
2032	Australia: ACT: Murrumbidgee River, 0.15 km u/s Uriarra Crossing (35°14.717'S, 148°57.135'E 440 m)
5569	Australia: ACT: Murrumbidgee River, 0.15 km u/s Uriarra Crossing (35°14.717'S, 148°57.135'E 440 m)
***Tasmanitachoides lutus* (Darlington)**	
1773	Australia: VIC: Jingalalla (Deddick) River, at Deddick Road, N. of Cobanandra, 525 m, (37.08S, 148.40E).
5582	Australia: VIC: Flat Rock Creek at highway B23, 256 m, 37.2835°S, 149.2223°E
***Tasmanitachoides* sp. “Lerderderg R**”	
1772	Australia: VIC: Lerderderg River, 6.8 km N. Bacchus Marsh, 135 m, (37.37'30"S, 144.25'24"E)
2029	Australia: VIC: Lerderderg River, 6.8 km N. Bacchus Marsh, 135 m, (37.37'30"S, 144.25'24"E)
5584	Australia: VIC: Lerderderg River, 6.8 km N. Bacchus Marsh, 135 m, (37.37'30"S, 144.25'24"E)
***Tasmanitachoides erwini* sp. nov.**	
5509	Australia: TAS: River Forth at C136, 126 m, 41.4712°S, 146.1366°E
5583	Australia: TAS: River Forth at C136, 126 m, 41.4712°S, 146.1366°E
5679	Australia: TAS: River Forth at C136, 126 m, 41.4712°S, 146.1366°E
**Tasmanitachoides cf. gerdi Baehr**	
2030	Australia: ACT: Murrumbidgee River, 0.15 km u/s Uriarra Crossing (35°14.717'S, 148°57.135'E 440 m)
5556	Australia: ACT: Murrumbidgee R. at Uriarra Crossing, 450 m, 35.2462°S, 148.9530°E
5575	Australia: ACT: Murrumbidgee R. at Angle Crossing, 598 m, 35.5825°S, 149.11°E
5676	Australia: ACT: Murrumbidgee R. at Uriarra Crossing, 450 m, 35.2462°S, 148.9530°E
***Tasmanitachoides* sp. “Angle Crossing #1**”	
5497	Australia: ACT: Murrumbidgee R. at Angle Crossing, 598 m, 35.5825°S, 149.1100°E
5578	Australia: ACT: Murrumbidgee R. at Angle Crossing, 598 m, 35.5825°S, 149.1100°E
5677	Australia: ACT: Murrumbidgee R. at Angle Crossing, 598 m, 35.5825°S, 149.1100°E
***Tasmanitachoides hobarti* (Blackburn)**	
5488	Australia: TAS: Lake St Clair, 741 m, 42.1121°S, 146.2051°E
5551	Australia: TAS: South Esk River at Avoca, 193 m, 41.7807°S, 147.7148°E
5552	Australia: TAS: Weld River NE Weldborough, 357 m, 41.1897°S, 147.9118°E
5554	Australia: TAS: Ringarooma River at Derby, 148 m, 41.1492°S, 147.8050°E
5579	Australia: TAS: Ringarooma River at Derby, 148 m, 41.1492°S, 147.8050°E
***Tasmanitachoides leai* (Sloane)**	
5507	Australia: VIC: Flat Rock Creek at highway B23, 256 m, 37.2835°S, 149.2223°E
5525	Australia: TAS: River Forth at C136, 126 m, 41.4712°S, 146.1366°E
5549	Australia: TAS: Ringarooma River at Derby, 148 m, 41.1492°S, 147.805°E
5557	Australia: TAS: South Esk River at Avoca, 193 m, 41.7807°S, 147.7148°E
5581	Australia: VIC: Flat Rock Creek at highway B23, 256 m, 37.2835°S, 149.2223°E
***Tasmanitachoides rufescens* Baehr**	
1993	Australia: ACT: Murrumbidgee River, 0.15 km u/s Uriarra Crossing (35°14.717'S, 148°57.135'E 440 m)
5577	Australia: ACT: Murrumbidgee R. at Angle Crossing, 600 m, 35.5803°S, 149.1109°E
***Tasmanitachoides* sp. “River Forth**”	
5555	Australia: TAS: River Forth at C136, 126 m, 41.4712°S, 146.1366°E
***Tasmanitachoides* sp. “Angle Crossing #2**”	
5550	Australia: ACT: Murrumbidgee R. at Angle Crossing, 598 m, 35.5825°S, 149.1100°E
5678	Australia: ACT: Murrumbidgee R. at Angle Crossing, 598 m, 35.5825°S, 149.1100°E
***Tasmanitachoides bicolor* Baehr**	
5568	Australia: QLD: Gayndah, Gray’s Waterhole
***Tasmanitachoides fitzroyi* (Darlington)**	
0762	Australia: QLD: Gayndah, Gray’s Waterhole
1575	Australia: QLD: Gayndah, Gray’s Waterhole
***Tasmanitachoides maior* Baehr**	
5508	Australia: ACT: Murrumbidgee R. at Angle Crossing, 600 m, 35.5803°S, 149.1109°E
5567	Australia: VIC Tambo River at Bruthen, 10 m, 37.7111°S, 147.8369°E
***Tasmanitachoides murrumbidgensis* (Sloane)**	
2031	Australia: ACT: Murrumbidgee River, 0.15 km u/s Uriarra Crossing (35°14.717'S, 148°57.135'E 440 m)
5553	Australia: VIC: Tambo River at Bruthen, 10 m, 37.7111°S, 147.8369°E
5564	Australia: ACT: Murrumbidgee R. at Angle Crossing, 598 m, 35.5825°S, 149.1100°E
5565	Australia: ACT: Murrumbidgee R. at Angle Crossing, 598 m, 35.5825°S, 149.1100°E
5566	Australia: VIC: Tambo River at Bruthen, 10 m, 37.7111°S, 147.8369°E
5571	Australia: VIC: Tambo River at Bruthen, 10 m, 37.7111°S, 147.8369°E
5572	Australia: VIC: Tambo River at Bruthen, 10 m, 37.7111°S, 147.8369°E
5754	Australia: ACT: Murrumbidgee R. at Angle Crossing, 600 m, 35.5803°S, 149.1109°E
***Tasmanitachoides* sp. “Tambo R**”	
5548	Australia: VIC: Tambo River at Bruthen, 10 m, 37.7111°S, 147.8369°E
5570	Australia: VIC: Tambo River at Bruthen, 10 m, 37.7111°S, 147.8369°E
5573	Australia: VIC: Tambo River at Bruthen, 10 m, 37.7111°S, 147.8369°E
5574	Australia: ACT: Murrumbidgee R. at Angle Crossing, 600 m, 35.5803°S, 149.1109°E
5576	Australia: ACT: Murrumbidgee R. at Angle Crossing, 600 m, 35.5803°S, 149.1109°E

Sites in 28S were chosen to be excluded from consideration using the modified GBLOCKS analysis (Talavera and Castresana 2007) present in Mesquite with the following options: minimum fraction of identical residues for a conserved position = 0.2, minimum fraction of identical residues for a highly-conserved position = 0.4, counting fraction within only those taxa that have non-gaps at that position, maximum number of contiguous non-conserved positions = 4, minimum length of a block = 4, and allowed fraction of gaps within a position = 0.5. No sites were excluded for 18S.

Maximum likelihood (ML) analysis was conducted for each gene individually using IQ-TREE version 1.6.12 (Nguyen et al. 2015), as orchestrated by Mesquite’s Zephyr package (Maddison and Maddison 2020b, c). The ModelFinder feature (Kalyaanamoorthy et al. 2017) within IQ-TREE was used to find the optimal character evolution models. The MFP model option was used for 28S and 18S, and the TESTMERGE option for the protein-coding genes. The TESTMERGE option sought the optimal partition of sites, beginning with codon positions in different parts. Fifty searches were conducted for the ML tree for each matrix.

In addition, analyses of a matrix formed by concatenation of all six gene fragments were conducted, with the TESTMERGE option also being used, beginning with each codon position for each gene as a separate part (thus, the analysis began allowing for up to 11 parts: three for each of the three protein-coding genes, as well as one for 28S and one for 18S). Fifty searches were conducted for the ML tree for each matrix; for bootstrap analyses, 500 replicates were performed. In addition, an equivalent ML search was conducted for a matrix formed by the concatenation of all gene fragments except COI.

## Data resources

Sequences have been deposited in GenBank with accession numbers MW291161 through MW291313. Aligned data for each gene as well as files containing inferred trees for each gene are available in Suppl. material 1, and have been deposited in the Dryad Digital Repository, https://doi.org/10.5061/dryad.8931zcrpv.

## Results

### Phylogeny

**Figure 5.**
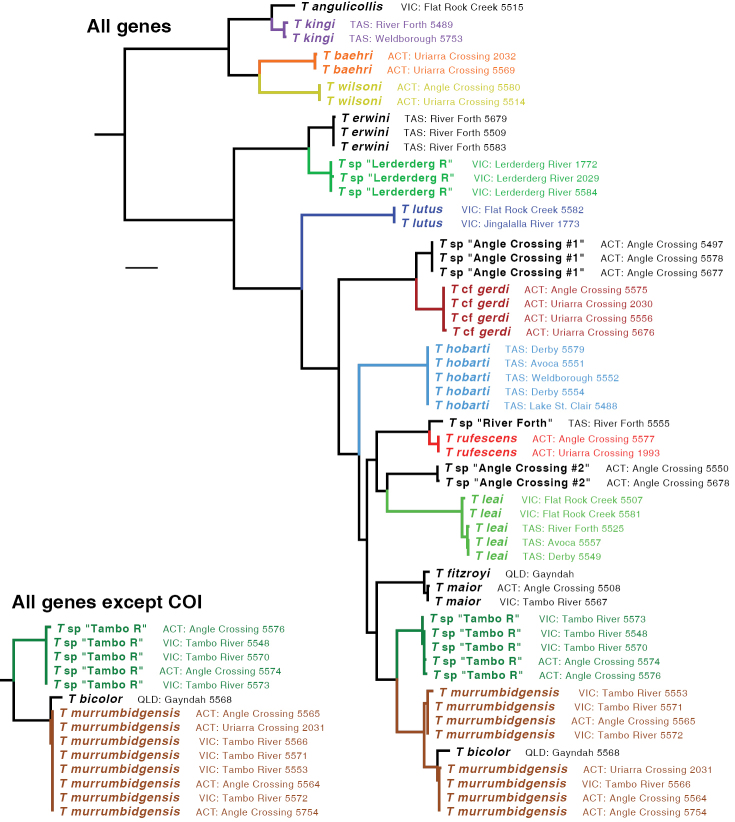
Maximum likelihood tree for the concatenated matrix of all gene fragments (main figure) and for the concatenated matrix of all genes except COI (inset; this shows only part of the tree). Branch length is shown proportional to relative divergence, as estimated by IQ-TREE; scale bar indicates 0.01 units. Outgroups (*Bembidarenas*, *Argentinatochoides*, and *Andinodontis*; Table 1) not depicted.

**Figure 6.**
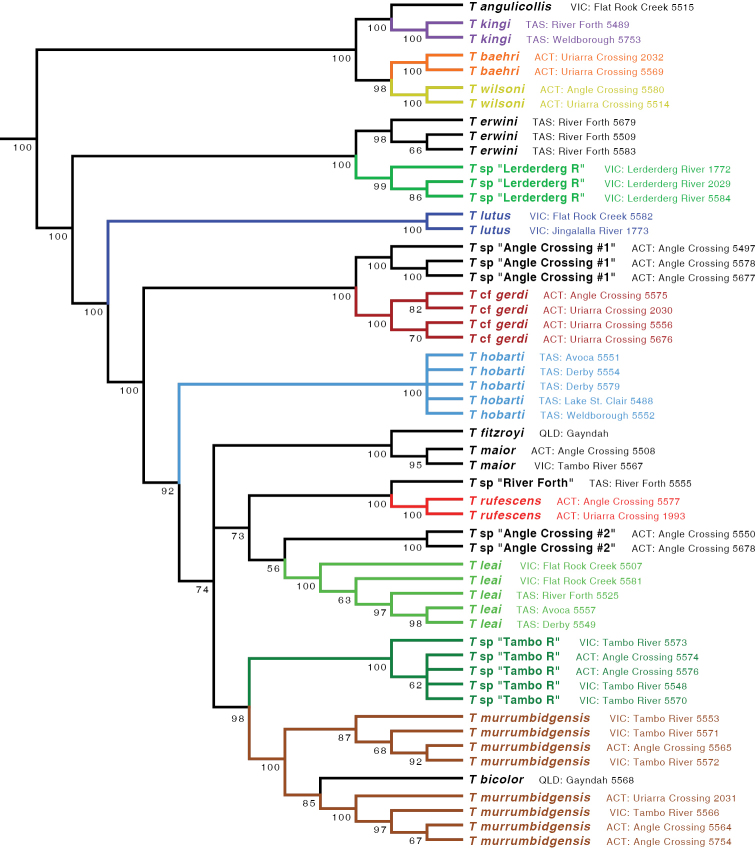
Majority rule consensus tree of maximum likelihood bootstrap trees for the concatenated matrix of all gene fragments. Numbers on branches indicate the percentage of bootstrap replicates containing that clade. Outgroups not depicted.

**Figure 7.**
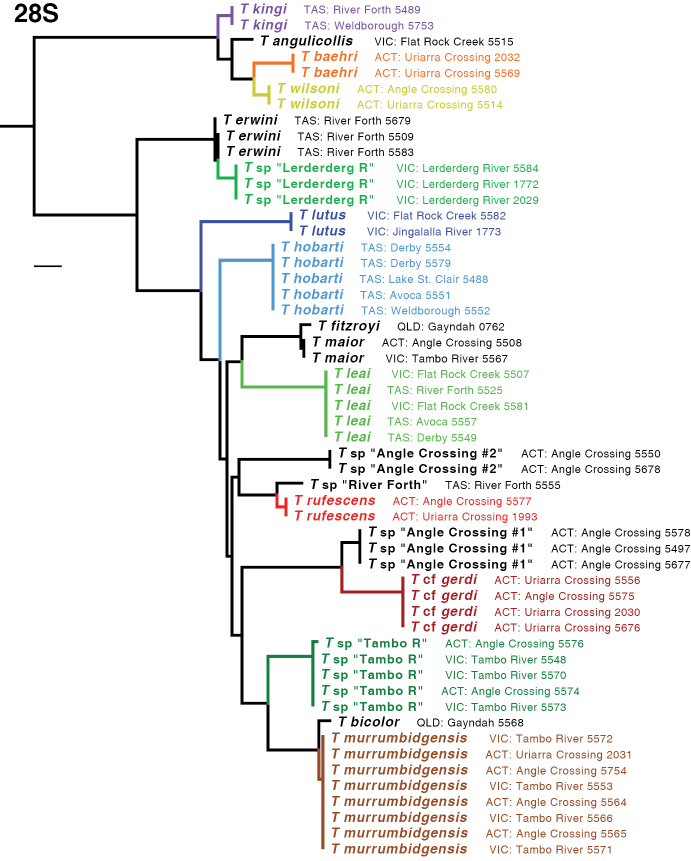
Maximum likelihood tree for 28S. Branch length is shown proportional to relative divergence, as estimated by IQ-TREE; scale bar indicates 0.01 units. Outgroups not depicted.

**Figure 8.**
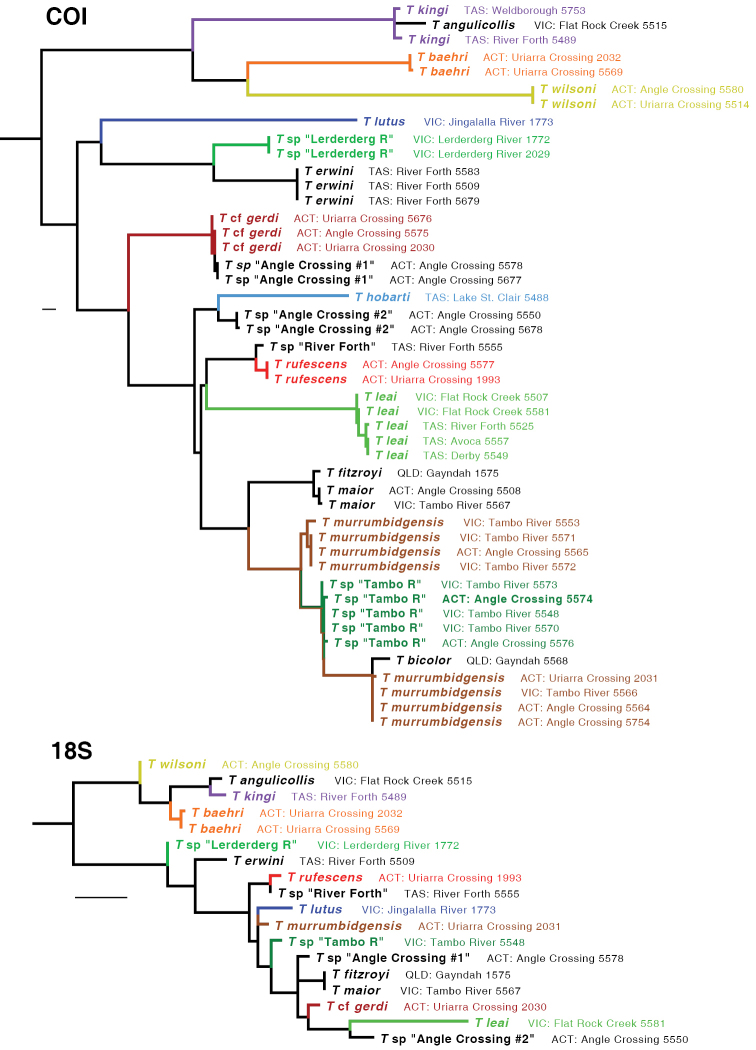
Maximum likelihood trees for COI and 18S. Branch length is shown proportional to relative divergence, as estimated by IQ-TREE; scale bars indicate 0.01 units. Outgroups not depicted.

**Figure 9.**
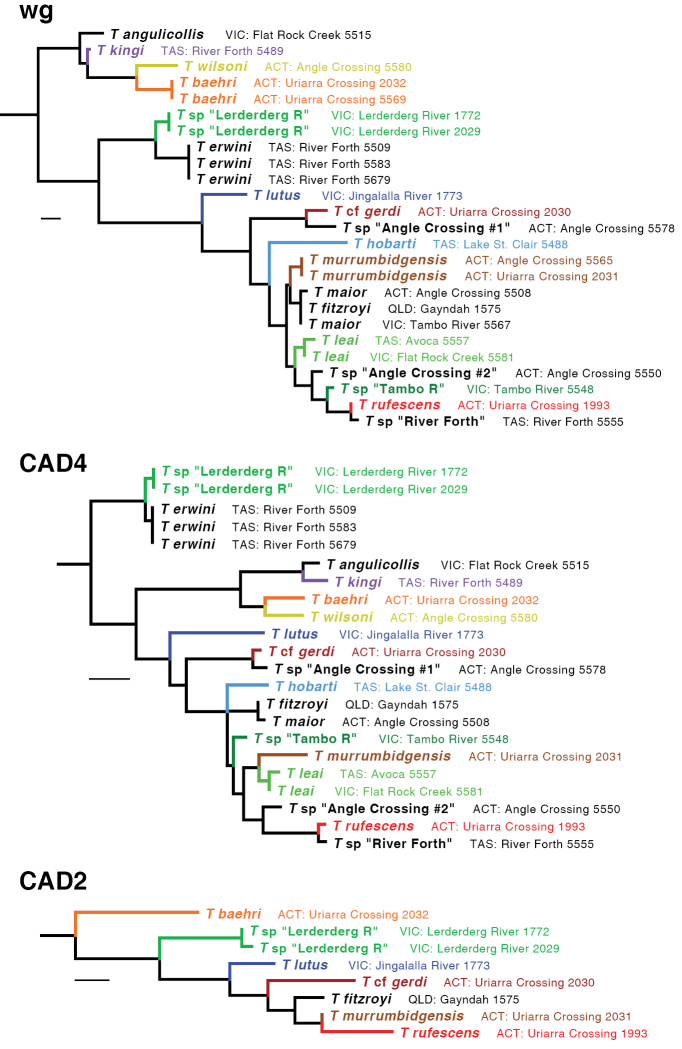
Maximum likelihood trees for wg, CAD4, and CAD2. Branch length is shown proportional to relative divergence, as estimated by IQ-TREE; scale bars indicate 0.01 units. Outgroups not depicted.

The ML tree for all six gene fragments combined is shown in Fig. 5. Many of the clades are well supported, as measured by bootstrap values (Fig. 6). The ML trees for individual genes are shown in Figs 7–9.

Based upon these analyses, the *kingi* group of *Tasmanitachoides* appears to be a clade that is sister to the remaining species. This result is supported by bootstrap values of 100%, and by ML trees for all gene fragments except for CAD4 (Figs 7–9). Within the remaining species, the two members sampled of the *wattsensis* group, *T. erwini* and *T.* sp. “Lerderderg R”, are sisters, as supported strongly by the combined analysis and by individual gene trees for 28S, COI, wg, and CAD4. This pair appears to be sister of all *Tasmanitachoides* except the *kingi* group; this is strongly supported by the concatenated analysis, but in individual genes only by 28S, wg, and CAD2. The morphologically distinctive *T. lutus* is isolated, with no near relatives. All remaining *Tasmanitachoides* (all but the *kingi* and *wattsensis* groups, as well as *T. lutus*) form a strongly supported clade; that clade is present in every gene tree except that of 18S (Figs 7–9). Within these remaining *Tasmanitachoides* there are some consistent results from gene to gene, in particular the close relationship between *T. fitzroyi* and *T. maior*, between T. cf. gerdi and *T.* sp. “Angle Crossing #1” (the two species of the *katherinei* group that were sampled), and between *T. rufescens* and *T.* sp. “River Forth”.

With one exception, for all species for which multiple specimens were sampled, the sequences of a species form a clade in the gene trees separate from specimens of other species. This is evident in the tree for 28S (Fig. 7), and for most of the COI tree (Fig. 8). The one exception is *T. murrumbidgensis*, which shows two distinctive clades in COI that are not each other’s sisters; in fact, one of those COI clades is in a clade with *T.* sp. “Tambo R” and *T. bicolor* (Fig. 8). Representatives of these two clades of *T. murrumbidgensis* were found together at both Angle Crossing (Murrumbidgee River) and along the Tambo River at Bruthen. The sequences in these two clades consistently differ at 13 of the 658 sites, for a divergence of approximately 2%. This is a very large difference in mitochondrial haplotypes within a species relative to divergences within other carabid species (e.g., Maddison 2008; Maddison and Cooper 2014; Maddison and Anderson 2016; Maddison and Sproul 2020). It is possible that there might be two species within what we call *T. murrumbidgensis*, but we can detect no morphological differences and there are no differences in other genes. Another possibility is that for one of these clades we have sequenced a nuclear copy (a numt, Thalmann et al. 2004), with the other clade representing the true mitochondrial gene, or they could represent the effects of *Wolbachia* infections (Smith et al. 2012), but we have no independent evidence supporting this. Whatever the nature of the sequences, the diversity within COI causes *T. murrumbidgensis* to appear as two separate clades within the multi-gene analyses (Fig. 5, main tree); these two separate clades are not evident if COI is excluded (Fig. 5, inset).

**Figure 10.**
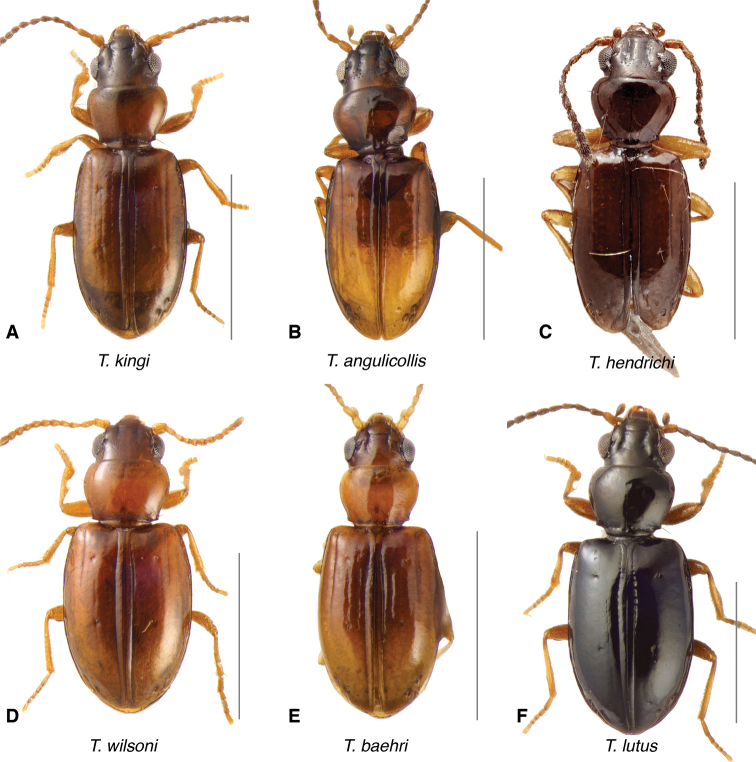
Adults of the *Tasmanitachoides kingi* species group as well as *T. lutus***A***T. kingi*, voucher V101468 **B***T. angullicollis*, voucher DNA5515. **C***T. hendrichi*, holotype. **D***T. wilsoni*, voucher V101470. **E***T. baehri*, voucher V101479. **F***T. lutus*, voucher V101462. Scale bars: 1.0 mm.

**Figure 11.**
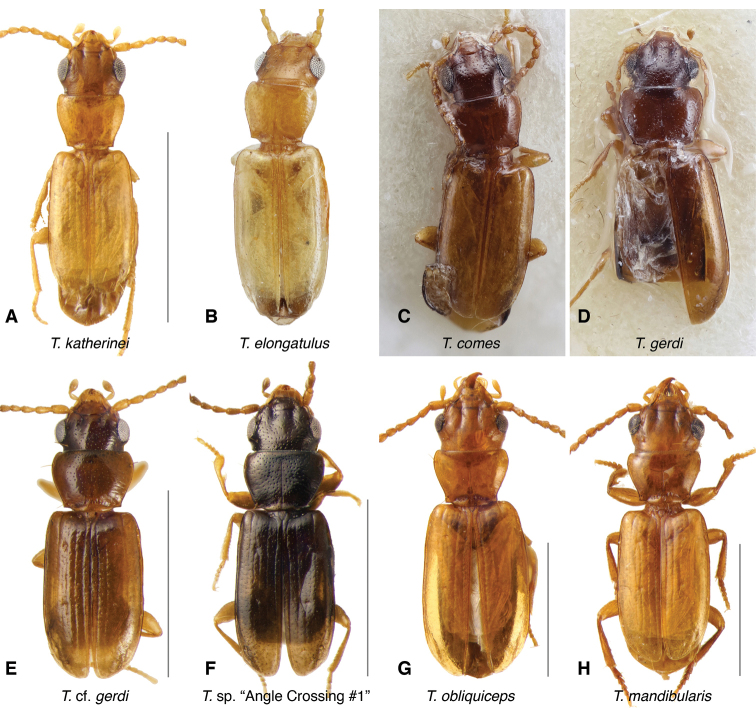
Adults of the *Tasmanitachoides katherinei* and *obliquiceps* species groups. **A***T. katherinei*, voucher V101468 **B***T. elongatulus*, voucher DNA5515 **C***T. comes*, holotype **D***T. gerdi*, holotype. **E**T. cf. gerdi, voucher DNA5676 **F***T.* sp. “Angle Crossing #1”, voucher DNA5677. **G***T. obliquiceps*, voucher V101477 **H***T. mandibularis*, voucher V101473. Scale bars: 1.0 mm.

**Figure 12.**
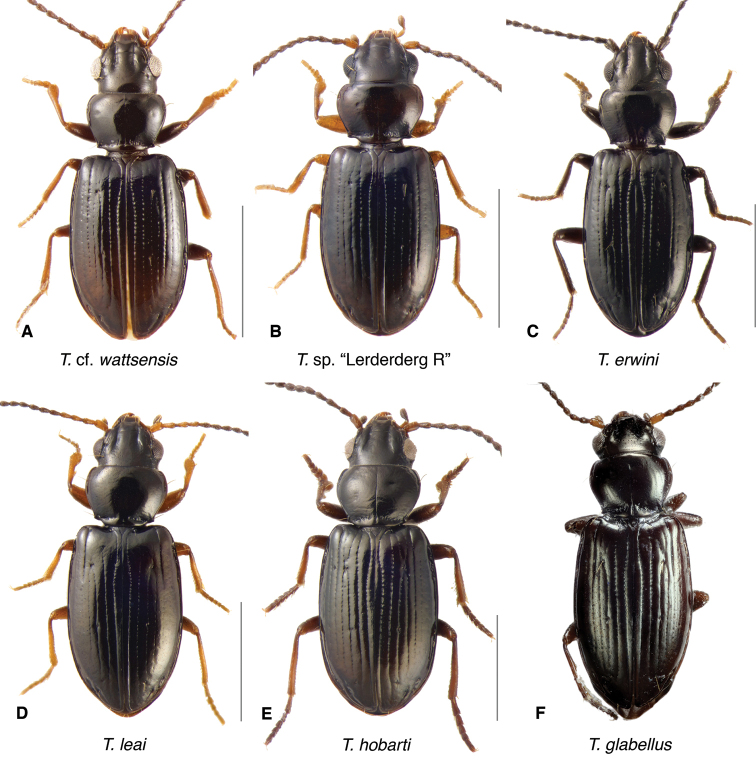
Adults of the *wattsensis* group and other *Tasmanitachoides***A***T. wattsensis*, voucher DNA5758; NSW: Leatherbarrel Creek u/s Alpine Way **B***T.* sp. “Lerderderg R”, voucher V101049; VIC: Lerderderg River, 6.8 km N. Bacchus Marsh **C***T. erwini*, voucher V101469 **D***T. leai*, voucher V101467 **E***T. hobarti*, voucher V101463 **F***T. glabellus*, paratype. Scale bars: 1.0 mm.

### Morphological variation

The known species of *Tasmanitachoides* vary in shape, form, and color (Figs 10–14). The elytral striation shows notable species-specific variation (Figs 15, 16), as do the structure of the clypeus and extent and structure of the frontal furrows of the head (Fig. 17), microsculpture (Fig. 18), and male genitalia (Figs 19, 20). We provide more details about this variation below in the Taxonomic treatment.

**Figure 13.**
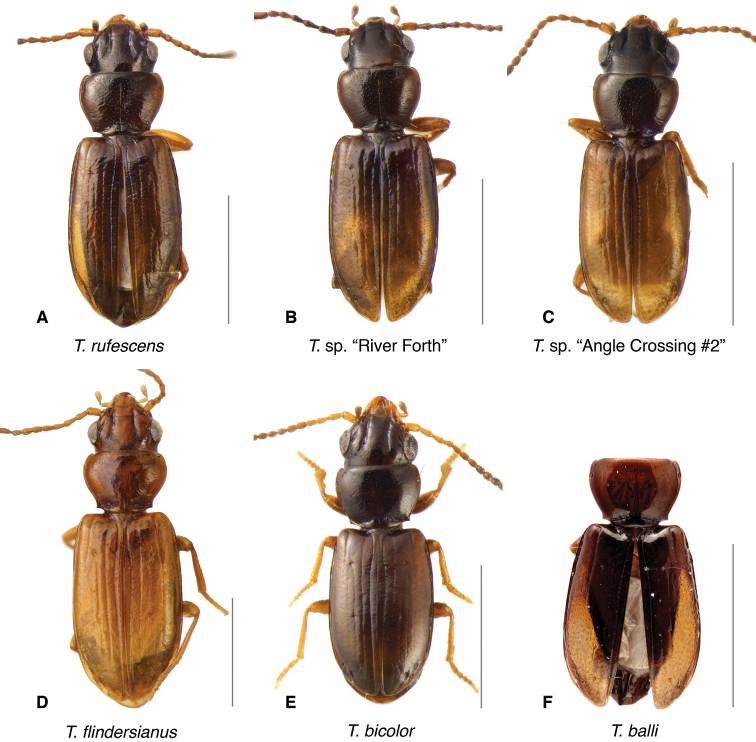
Adults of *Tasmanitachoides***A***T. rufescens*, voucher V101478 **B***T.* sp. “River Forth”, voucher DNA5555 **C***T.* sp. “Angle Crossing #2”, voucher DNA5678 **D***T. flindersianus*, paratype **E***T. bicolor*, voucher V101472 **F***T. balli*, holotype. Scale bars: 1.0 mm.

**Figure 14.**
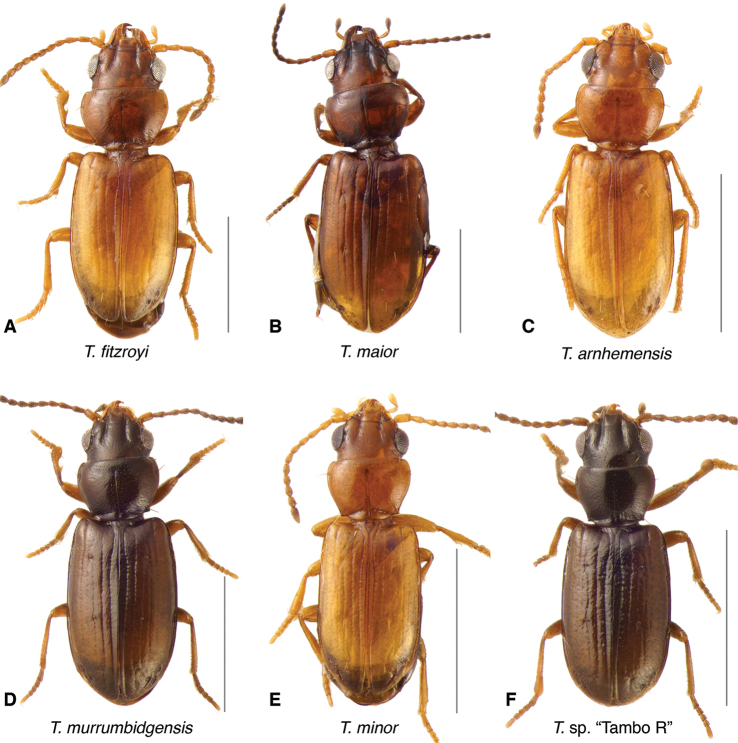
Adults of the *Tasmanitachoides fitzroyi* species group **A***T. fitzroyi*, voucher V101471 **B***T. maior*, voucher DNA5508 **C***T. arnhemensis*, voucher V10476 **D***T. murrumbidgensis*, voucher V101464 **E***T. minor*, voucher V101474 **F***T.* sp. “Tambo R”, voucher V101465. Scale bars: 1.0 mm.

**Figure 15.**
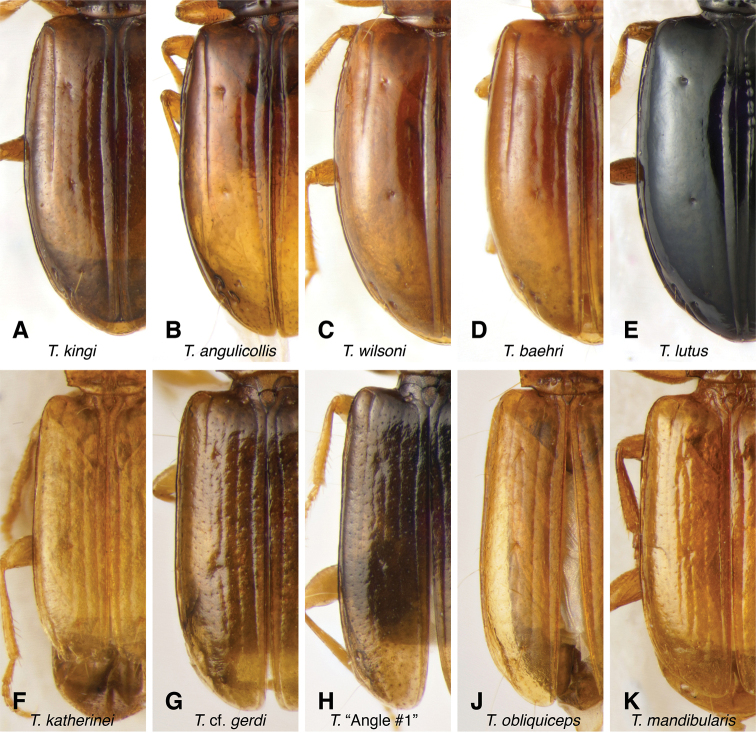
Left elytron of *Tasmanitachoides***A***T. kingi*, voucher V101468 **B***T. angulicollis*, voucher DNA5515 **C***T. wilsoni*, voucher V101470 **D***T. baehri*, voucher V101479 **E***T. lutus*, voucher V101462 **F***T. katherinei*, voucher V101475 **G**T. cf. gerdi, voucher DNA5676 **H***T.* sp. “Angle Crossing #1”, voucher DNA5677 **J***T. obliquiceps*, voucher V101477 **K***T. mandibularis*, voucher V101473.

**Figure 16.**
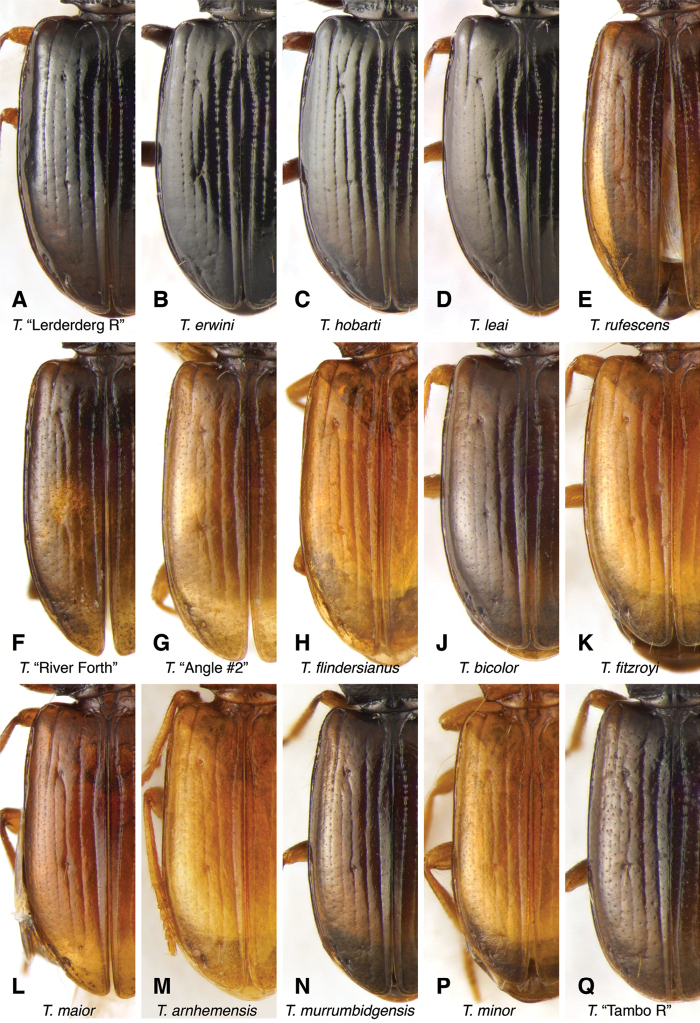
Left elytron of *Tasmanitachoides***A***T.* sp. “Lerderderg R”, voucher V101049 **B***T. erwini*, voucher V101469 **C***T. hobarti*, voucher V101463 **D***T. leai*, voucher V101467 **E***T. rufescens*, voucher V101478 **F***T.* sp. “River Forth”, voucher DNA5555 **G***T.* sp. “Angle Crossing #2”, voucher DNA5678 **H***T. flindersianus*, paratype **J***T. bicolor*, voucher V101472 **K***T. fitzroyi*, voucher V101471 **L***T. maior*, voucher DNA5508 **M***T. arnhemensis*, voucher V101476. **N***T. murrumbidgensis*, voucher V101464 **P***T. minor*, voucher V101474 **Q***T.* sp. “Tambo R”, voucher V101465.

### Taxonomic treatment

Diagnoses and descriptions of the genus are provided by Erwin (1972) and Baehr (2013). We are aware of 32 species of *Tasmanitachoides*: 25 previously described, two described in this paper, and five additional species whose descriptions await future research. Based upon the phylogenetic results and morphological variation, most species can be tentatively arrayed into six species groups, as follows:

*kingi* group

*T. kingi* (Darlington)

*T. angulicollis* Baehr

*T. hendrichi* Baehr

*T. wilsoni* (Sloane)

*T. baehri* sp. nov.

*wattsensis* group

*T. wattsensis* (Blackburn)

*T.* sp. “Lerderderg R”

*T. erwini* sp. nov.

*lutus* group

*T. lutus* (Darlington)

*katherinei* group

*T. katherinei* Erwin

*T. elongatulus* Baehr

*T. comes* Baehr

*T. gerdi* Baehr

*T.* sp. “Angle Crossing #1”

*obliquiceps* group

*T. obliquiceps* (Sloane)

*T. mandibularis* Baehr

*fitzroyi* group

*T. fitzroyi* (Darlington)

*T. maior* Baehr

*T. arnhemensis* Erwin

*T. murrumbidgensis* (Sloane)

*T. minor* Baehr

*T.* sp. “Tambo R”

*T. bicolor* Baehr

unplaced to group

*T. hobarti* (Blackburn)

*T. glabellus* Baehr

*T. leai* (Sloane)

*T. hackeri* Baehr [likely a synonym of *T. leai*]

*T. balli* Baehr

*T. rufescens* Baehr

*T.* sp. “River Forth”

*T.* sp. “Angle Crossing #2”

*T. flindersianus* Baehr

The placement of species into groups may change once more species are better known, including those we have not sampled for DNA.

### Identification of species of *Tasmanitachoides*

Species of *Tasmanitachoides* are currently very difficult to identify using morphological characteristics, in part because they are small, and as the known external differences between many species are subtle. In addition, although the internal sac of the male aedeagus has a complex pattern of sclerites, and thus could be a very valuable source of characters for identification, genitalic variation is not well documented or understood. One difficulty with comparing male genitalia is that the internal sac sclerites are oriented in a plane that is nearly edge-on in the standard left lateral view. This causes them to appear very differently as a function of slight differences in the orientation of the genitalia (compare, for example, Fig. 20B to Fig. 20C), causing interpretation of sclerites in the standard left lateral view troublesome. In contrast, a ventral view (e.g., Fig. 19) shows patterns much less sensitive to slight differences in angle. As previously published images of male genitalia (e.g., Erwin 1972; Baehr 1990) are of the standard left lateral view, the genitalia of all species will need to be re-examined to provide a more robust understanding of variation.

The key we present below is only an incremental improvement on Baehr’s (2010; 2013). We began with his key and modified it to include the two new species we describe, as well as some (but not all) of the undescribed species of which we are aware. We have not included *T.* sp. “Lerderderg R”, *T.* sp. “Angle Crossing #1”, and *T.* sp. “River Forth”. We have included *T. balli*, although with some doubt, as the only known specimen is now missing its head.

Based upon our examination of specimens of all known species, we have removed some of the inconsistencies in the key, simplified it, and changed its structure somewhat. However, we view this as a provisional key. Although we have previously seen specimens of all known species, for some of them (e.g., *T. glabellus*, *T. comes*, *T. gerdi*) we modified the key without those specimens at hand, and depended upon our notes and photographs of the primary types, as well as Martin Baehr’s papers. In addition, the variation within many species is not yet known, as there is limited material available (ten of the described species are known from fewer than five specimens). For example, Baehr’s key uses size to separate *T. maior* from other species, noting that the only specimen he knew was 2.9 mm in length; however, based upon our somewhat larger sample (we have measured seven specimens) the holotype is at the upper end of the size range, with some specimens as small as 2.44 mm in length, overlapping in length with related species. We suspect that the sizes given in the key for many species will need to be modified once more material is examined. The same will likely be true for color, as we have seen more variation in color in our large samples of some species than Martin Baehr had seen in his smaller samples. In addition, the geographic distributions mentioned in the key should be viewed with suspicion, as the ranges of species are very poorly known.

In Baehr’s keys, the couplet which divides *Tasmanitachoides* into the largest two groups is that which focuses on whether or not the clypeus is “distinctly impressed anteriorly”. We find this character difficult to ascertain, with many specimens appearing ambiguous, in part because of the more or less continuous variation in this trait across *Tasmanitachoides* species. For this reason we have replaced this couplet with one that focuses instead on a related trait, the presence or absence of tubercles on the anterior lateral corners of the clypeus, with associated modifications to other regions of the clypeus; this latter character is easier to judge.

### Provisional key to all described and a few undescribed species of *Tasmanitachoides*

**Table:** 

1	Only the sutural stria distinct, others completely effaced or almost so (Fig. 10F)	**(*lutus* group) *T. lutus* (Darlington)**
–	Elytra with at least stria 5 impressed anteriorly, other striae superficial to deeply impressed	**2**
2	Anterior lateral corners of the clypeus raised, tuberculate, such that the central region of the anterior half of the clypeus is distinctly lower than the lateral regions (Fig. 17L, M; best observed with the anterior portion of the head tilted up, and under diffuse lighting or a ring light). Each raised lateral tubercle is bounded laterally by a steep drop to the smooth frontal furrow, which is relatively straight in its anterior half and extends to the front corner of the clypeus	***fitzroyi* group, 3**
–	Anterior lateral regions of clypeus not obviously tuberculate; the central region is thus convex, flat, or only slightly concave (Fig. 17A–K). Frontal furrow not as straight, deep, or smooth on clypeus	**8**
3	Body larger and wider (Fig. 14A, B), ≥ 2.4 mm long (doubtful species under both couplets). Pronotum broad, convex. At least elytra rufous or rufo-testaceous	**4**
–	Body smaller and narrower (Fig. 14C–F), < 2.3 mm long. Pronotum variable; color variable.	**5**
4	Color uniformly rufous or rufo-piceous; apical antennomeres infuscated. ACT, NSW, VIC	***T. maior* Baehr**
–	Color pale with rufo-testaceous forebody, elytra at apex testaceous (Fig. 14A); antennae and palpi yellow; frontal furrows distinctly divergent. Northern QLD, northern NT, northern WA	***T. fitzroyi* (Darlington)**
5	Forebody reddish to reddish-testaceous, elytra testaceous. NT, WA	**6**
–	Either completely piceous or dark reddish, or forebody dark piceous and elytra dark reddish with piceous borders, suture, base, and apex. VIC, ACT, NSW	**7**
6	Pronotum broader, lateral margin of pronotum strongly rounded, with short, straight region just in front of the projected hind angle; second to fourth elytral striae less impressed. Body larger and wider, 1.9–2.15 mm long. Central and northern NT, northern WA.	***T. arnhemensis* Erwin**
–	Lateral border of pronotum distinctly sinuate in front of the right-angled, but non-projected hind angle; second to fourth elytral striae more impressed. Body smaller and narrower, 1.65–1.95 mm long. Northern WA, north of Great Sandy Desert.	***T. minor* Baehr**
7	Body more convex, broader, especially the pronotum, which is more evidently wider than the head (maximum width of prothorax/width of head across eyes 1.12–1.15, n = 5). Lateral margins of pronotum more rounded, especially around anterior lateral seta. Body size in general larger, 1.87–2.17 mm, most specimens > 1.90 mm.	***T. murrumbidgensis* (Sloane)**
–	Body flatter, narrower; pronotum only very slightly wider than head (maximum width of prothorax/width of head across eyes 1.04–1.08, n 5). Lateral margins of pronotum less rounded, straighter. Body size in general smaller, 1.64–1.97 mm, most specimens < 1.90 mm	***T.* sp. “Tambo R**”
8	Head very large, with large, elongate mandibles (Fig. 11G, H); eyes small, depressed, with well-developed temples, posterior supraorbital seta situated far behind eye; pronotum trapezoid, widest shortly behind anterior angles; color testaceous	***obliquiceps* group, 9**
–	Head of a relative size more typical for a carabid, with shorter, less protruding mandibles; eyes larger, more protruded, temples, if evident, small; posterior supraorbital seta situated immediately at posterior border of eye; pronotum laterally more rounded, widest far behind anterior angles; color variable	**10**
9	Larger species, body length > 2 mm; pronotum wider, ratio width/length > 1.35; elytra longer, ratio length/width > 1.75; pronotum impunctate; pilosity on pronotum and elytra barely visible even at high magnification. NSW, QLD	***T. obliquiceps* (Sloane)**
–	Smaller species, body length < 1.85 mm; pronotum narrower, ratio width/length < 1.32; elytra shorter, ratio length/width < 1.68; pronotum finely punctate; pilosity on pronotum and elytra distinct, erect. WA	***T. mandibularis* Baehr**
10	Third and fourth elytral striae absent, or barely recognizable (except in some species as shallow, broad, impunctate grooves, Fig. 18A), without punctures. Frontal furrows shallow and short, indistinct for most of their length	***kingi* group, 11**
–	Third and fourth elytral striae present, with at least small punctures, although sometimes superficial. Frontal furrows variable	**15**
11	Pronotum narrow, much narrower than the elytra at the shoulders (Fig. 10E), and approximately the same width as head; elytral intervals 2–5 convex, with striae evident as broad, shallow, impunctate grooves between them (Figs 15D, 18A). On head a groove extends from the anterior supraorbital puncture anteriad and mediad to approximately halfway toward the frontal furrow (Fig. 17B). Hind angle of pronotum obtuse	***T. baehri* sp. nov.**
–	Pronotum closer to the width of the elytra at the shoulders (Fig. 10A–D), and more evidently wider than the head; intervals 3 and 4 not convex, striae 2 either absent, or, if present, composed of a narrow striation rather than a broad groove. If there is a groove extending from the anterior supraorbital puncture, it is very short (Fig. 17A). Hind angle of pronotum obtuse to acute	**12**
12	Body short and convex (Fig. 10D); elytra considerably less than 1.5 × longer than wide; pronotum wide, base (at hind angles) as wide as apex, hind angle greater than 90°, laterally not projected. Body orange or orange-brown. Second stria effaced	***T. wilsoni* (Sloane)**
–	Body longer and narrower, less convex; elytra more than 1.5 × longer than wide; pronotum narrower, base (at hind angles) considerably narrower than apex, hind angle acute, laterally projected. Body orange-brown or darker. Second stria effaced or present	**13**
13	Eyes less protruded, temples perceptible; hind angle of pronotum approximately 90°, less acute and projected. Most specimens with body infuscated. TAS	***T. kingi* (Darlington)**
–	Eyes more protruded, temples reduced; hind angle of pronotum acute, < 90°, laterally distinctly projected. Most specimens with body orange or orange-brown. VIC, NSW	**14**
14	Prothorax wider, ratio width/length > 1.25; elytra shorter and wider, ratio length/width 1.55; finest traces of striae still visible between first and fifth stria	***T. angulicollis* Baehr**
–	Prothorax narrower, ratio width/length <1.15; elytra longer and narrower, ratio length/width l.70; virtually no traces of striae visible between first and fifth striae	***T. hendrichi* Baehr**
15	Frontal furrows short (Fig. 17D, E); body flatter, elongate, narrow; size small, 1.5–l.7 mm; color testaceous to light piceous. NT, WA, QLD, NSW, ACT	***katherinei* group, 16**
–	Frontal furrows longer (Fig. 17G–M); body more convex, wider; size larger, 1.7–2.6 mm; color dark reddish to black, or reddish with distinctly paler elytra	**19**
16	Entire dorsal surface with distinct isodiametric microsculpture; color testaceous, in some specimens head and prothorax slightly darker than elytra; elytra generally shorter, ratio length/width of elytra < l.65. Northern NT, northern WA, northern QLD, northeastern NSW	***T. katherinei* Erwin**
–	Dorsal surface with at most indistinct, superficial microsculpture, in particular elytra which are shiny with microsculpture almost effaced; color various; elytra generally longer, ratio length/width > 1.70. Northern WA, northern QLD	**17**
17	Frontal furrows attaining but the anterior third of the eyes, ended abruptly (Fig. 17D); pronotum wider, ratio width/length 1.33, barely sinuate in front of hind angles, with wider base compared with apex, ratio width of apex/width of base 1.10 (Fig. 11D).	***T. gerdi* Baehr**
–	Frontal furrows attaining mid-level of the eyes, ended less abruptly (Fig. 17E); pronotum narrower, ratio width/length ≤ 1.26, distinctly sinuate in front of hind angles, with narrower base compared with apex, ratio width of apex/width of base > 1.18 (Fig. 11C).	**18**
18	Color darker, head and pronotum dark reddish to reddish-piceous, elytra reddish (Fig. 11C); elytra shorter, ratio length/width 1.76, striae 2 and 3 less impressed than striae 1 and 4	***T. comes* Baehr**
–	Color paler, head and pronotum pale reddish, elytra pale yellow (Fig. 11B); elytra longer, ratio length/width > 1.81, striae 2 and 3 as deeply impressed as striae 1 and 4. Northwestern QLD & northeastern NT	***T. elongatulus* Baehr**
19	Pronotum constricted posteriad such that the hind margin is notably narrower than width at widest point, with lateral margin distinctly sinuate (Fig. 13D); hind angles rectangular or acute. Elytra relatively flat. Body color uniformly reddish or dark reddish. Body size large, 2.6–2.8 mm	***T. flindersianus* Baehr**
–	Pronotum less constricted, with sides less sinuate (Figs 12, 13A–C, E–F). Elytra flat or convex. Color of at least head and pronotum generally darker. Body size generally smaller, < 2.6 mm	**20**
20	First elytral stria straighter, less abruptly sinuate (Fig. 16E–G, J), with or without distinct punctures in the anterior half. Fifth elytral interval distinctly impressed in the anterior fifth to third of the elytra, abruptly less distinct behind that point (Fig. 16E–G, J). Body relatively flat (Fig. 13A–C), except for *T. bicolor* which is slightly convex (Fig. 13E). With distinct microsculpture on the elytra, and thus the surface is duller. Body color either almost uniformly reddish or dark reddish, or piceous with disk of each elytron contrastingly lighter, or body uniformly piceous (if body uniformly piceous, then length < 2.0 mm). Body size generally smaller, 1.7–2.4 mm	**21**
–	First elytral stria abruptly sinuate, very close to the suture in the anterior fifth or fourth, at which point it abruptly bends away from the suture (Fig. 16A–D); with distinct punctures in the anterior half. First interval is at its widest at approximately the one-third point, as wide or wider than the second interval, and from that point back it gradually narrows. Fifth elytral interval not abruptly shallower within the first third of the elytra, well-impressed for at least the first half (Fig. 16A–C), except for *T. leai* (which is convex, dark, and shiny, Fig. 12D). Body convex (except for *T. erwini*, which is very dark, Fig. 12C). Microsculpture on elytra more effaced, and thus the surface is shinier. Body color uniformly dark piceous to black, or piceous with elytra slightly (not contrastingly) lighter (Fig. 12). Body size in general larger, 2.1–2.6 mm	**24**
21	Eyes large, more protruded, temples almost wanting (Fig. 17G–J, L, M); forebody piceous, elytra lighter on disk, color of body and antennae rather contrasting; pronotum less narrowed towards base. Body size in general smaller, 1.78–2.05 mm	**22**
–	Eyes smaller, less protruded, temples more evident, oblique (Fig. 17K); upper surface completely dark reddish to light piceous, elytra at most slightly lighter, color of body and antennae little contrasting; pronotum rather narrowed towards base. Body size in general larger, 2.0–2.3 mm	***T. rufescens* Baehr**
22	Body more convex, especially the pronotum. Pronotum with lateral margins more rounded. Apical antennomeres darker, such that antennomere 9 is much darker than antennomere 4. QLD	**23**
–	Body flatter. Pronotum with lateral margins straighter. Apical antennomeres only slightly darker than basal antennomeres, such that antennomere 9 is only slightly darker than antennomere 4. ACT	***T.* sp. “Angle Crossing #2**”
23	Color uniformly dark piceous; elytra slightly shorter and wider, ratio length/width approximately 1.65; microsculpture on pronotum and elytra much more superficial; punctation of intervals very fine, less distinct. Northern QLD, Iron Range, mid Cape York Peninsula	***T. balli* Baehr**
–	Forebody piceous, elytra distinctly lighter on disk; elytra slightly longer and narrower, ratio length/width approximately 1.75; microsculpture on pronotum and elytra much more distinct; punctation of intervals coarser, distinct. Northeastern QLD, south of Cape York Peninsula	***T. bicolor* Baehr**
24	Fifth elytral stria abruptly ended behind anterior third (Fig. 16D); pronotum and elytra markedly convex. TAS, VIC, QLD	***T. leai* (Sloane) and *T. hackeri* Baehr**
–	Fifth elytral stria distinct even in apical half (Fig. 16A–C); pronotum and elytra flatter. TAS, SA, VIC, NSW, QLD	**25**
25	Third and fourth elytral striae at halfway point of elytra much less impressed than second and fifth; sixth stria absent or extremely faint	***wattsensis* group, 26**
–	Third and fourth elytral striae more less as impressed as second; sixth stria distinct, consisting of evident punctures	**27**
26	Legs darker, with femur piceous, tibia at least mostly piceous, and tarsi at least infuscated if not piceous; all antennomeres piceous, including first antennomere. Body less convex, dorsal surface flatter. TAS	***T. erwini* sp. nov.**
–	Legs paler, with femur rufo-testaceous or infuscated, tibia at most slightly infuscated, and tibia rufo-testaceous or testaceous; first antennomere distinctly paler than others. Body relatively convex. VIC, southern NSW	***T. wattsensis* (Blackburn)**
27	Occurring in TAS	***T. hobarti* (Blackburn)**
–	Occurring in QLD	***T. glabellus* Baehr**

Below we provide notes about some of the species of *Tasmanitachoides*, as well as descriptions of *T. baehri*, sp. nov., and *T. erwini*, sp. nov.

#### Tasmanitachoides baehri sp. nov.

Taxon classificationAnimaliaColeopteraCarabidae

E93DED16-DE9F-58DD-BCEA-D5E300B8C612

http://zoobank.org/D4F6DB85-62FE-47DA-A67E-91C2FB54BA2B

**Figure 17.**
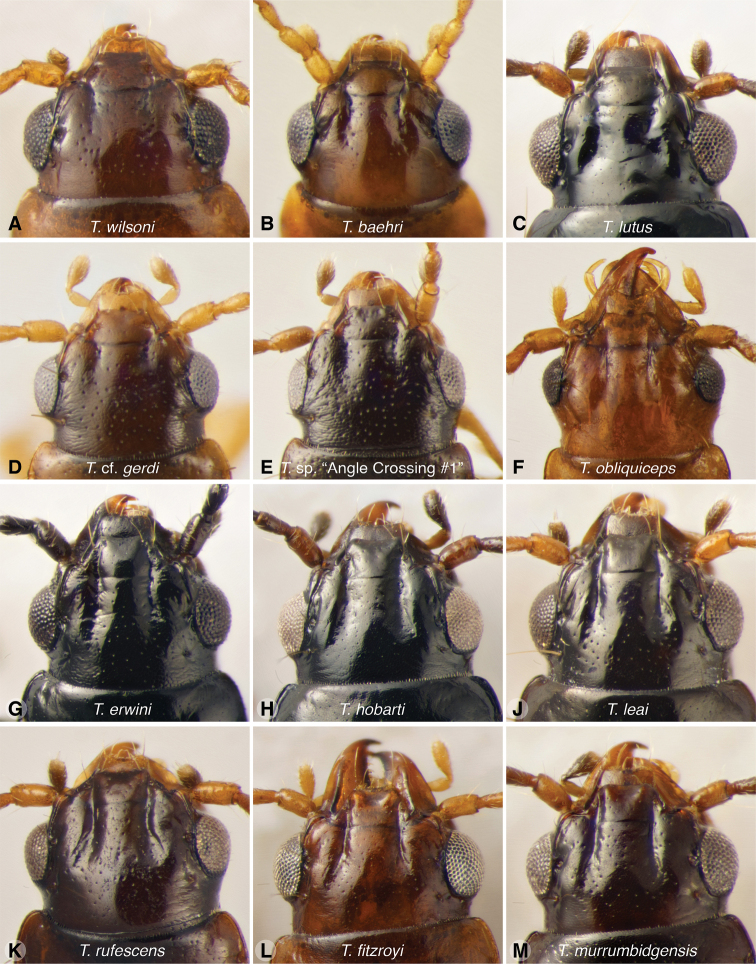
Dorsal view of head of *Tasmanitachoides* adults **A***T. wilsoni*, voucher V101470 **B***T. baehri*, voucher V101479 **C***T. lutus*, voucher V101462 **D**T. cf. gerdi, voucher DNA5676 **E***T.* sp. “Angle Crossing #1”, voucher DNA5677 **F***T. obliquiceps*, voucher V101477 **G***T. erwini*, voucher V101469 **H***T. hobarti*, voucher V101463 **J***T. leai*, voucher V101467 **K***T. rufescens*, voucher V101478 **L***T. fitzroyi*, voucher V101471 **M***T. murrumbidgensis*, voucher V101464.

**Figure 18.**
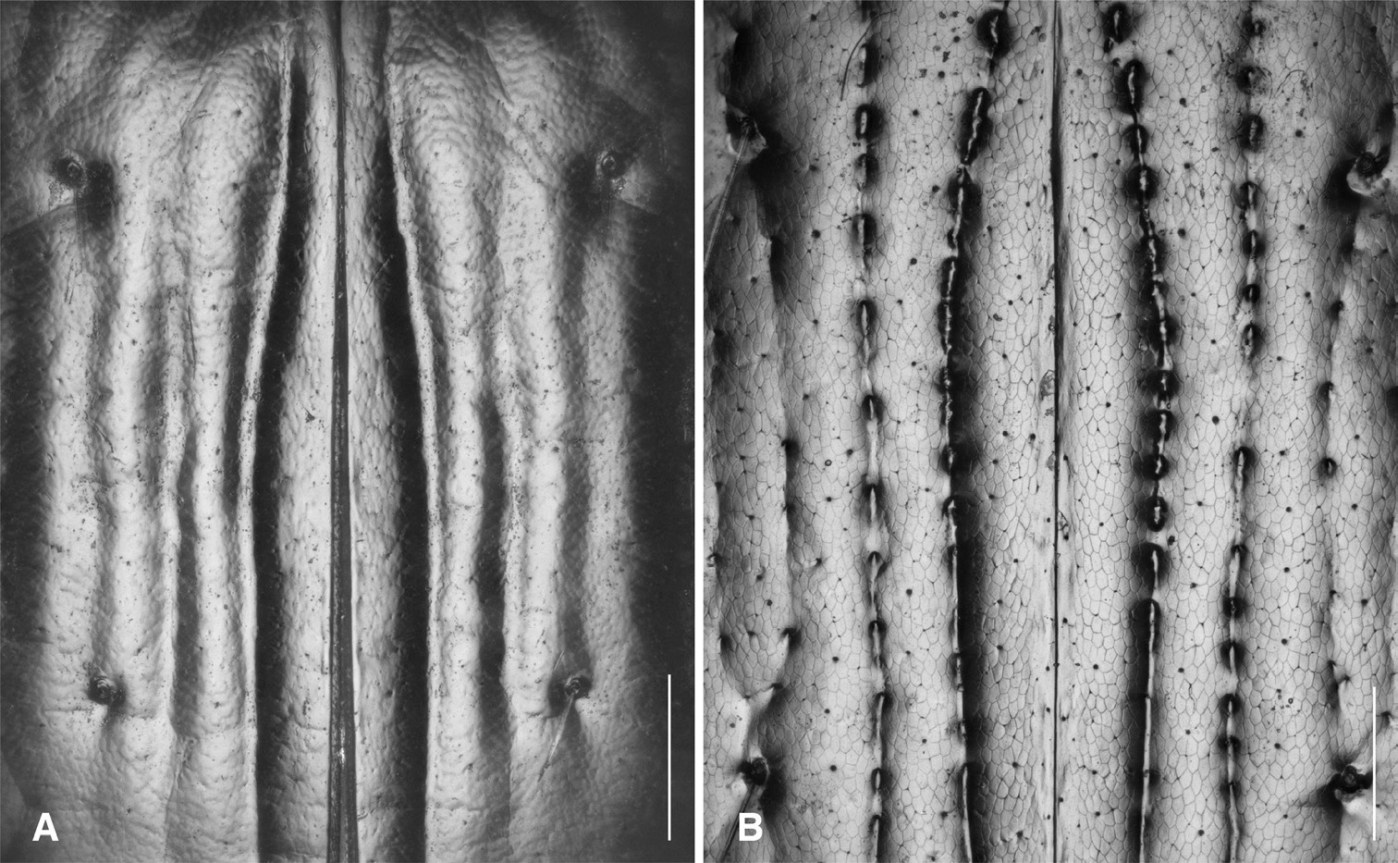
Elytral microsculpture of *Tasmanitachoides*, dorsal view **A***T. baehri*, voucher V101484 **B***T. erwini*, voucher V101483. Scale bar: 100 µm.

**Figure 19.**
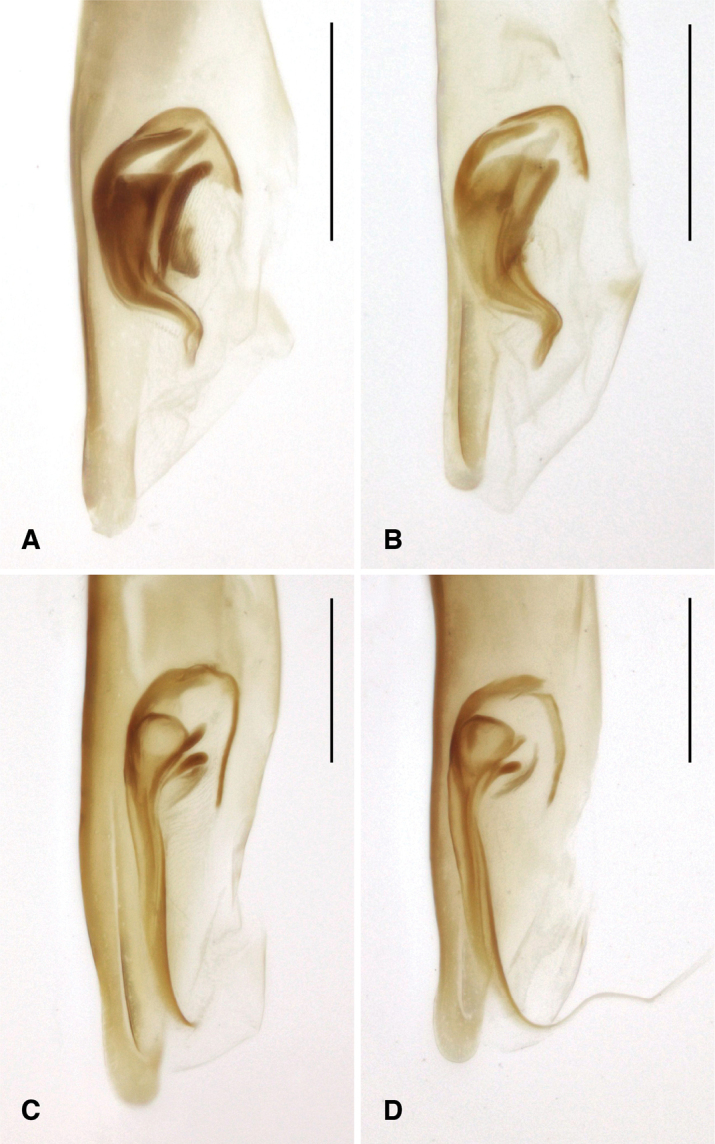
Aedeagus of *Tasmanitachoides*, ventral view **A***T. baehri*, voucher DNA5569 (holotype) **B***T. wilsoni*, voucher DNA5514 **C***T. erwini*, voucher V101481 **D***T.* sp. “Lerderderg R”, voucher DNA2029. Scale bars: 100 µm.

**Figure 20.**
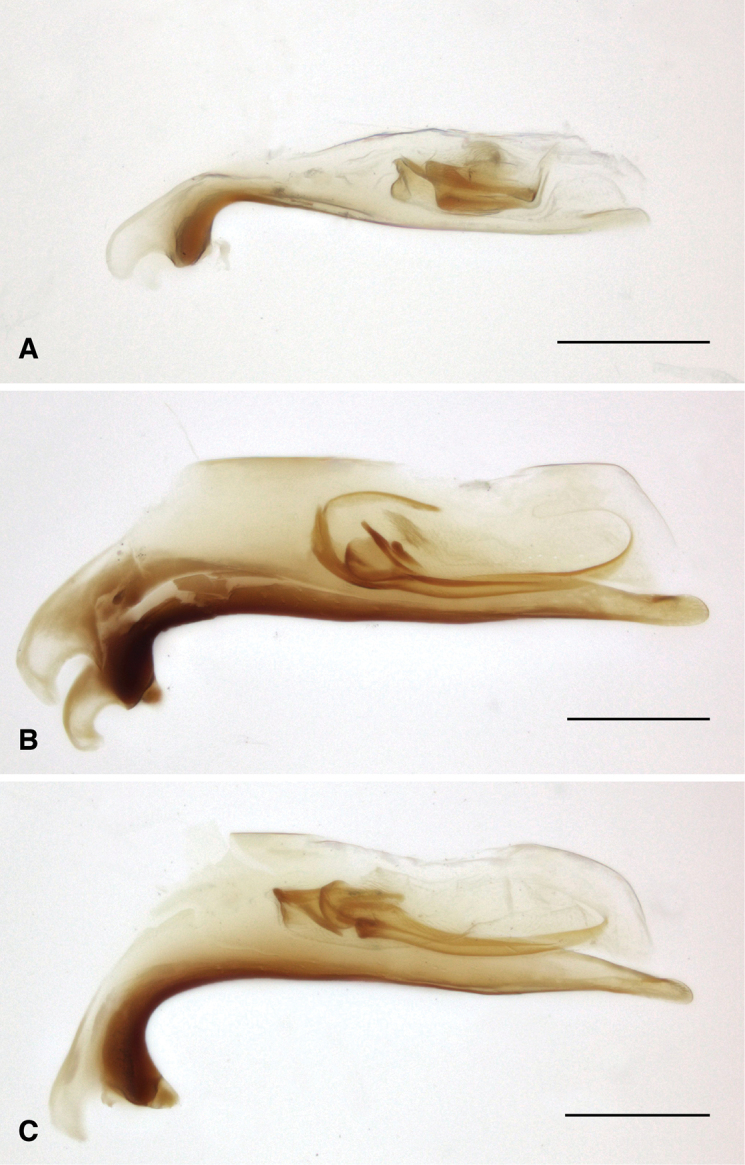
Aedeagus of *Tasmanitachoides*, left lateral view **A***T. baehri*, voucher DNA2032 **B***T. erwini*, voucher DNA5583 **C***T. erwini*, voucher DNA5509 (holotype). Scale bar: 100 µm.

**Figure 21.**
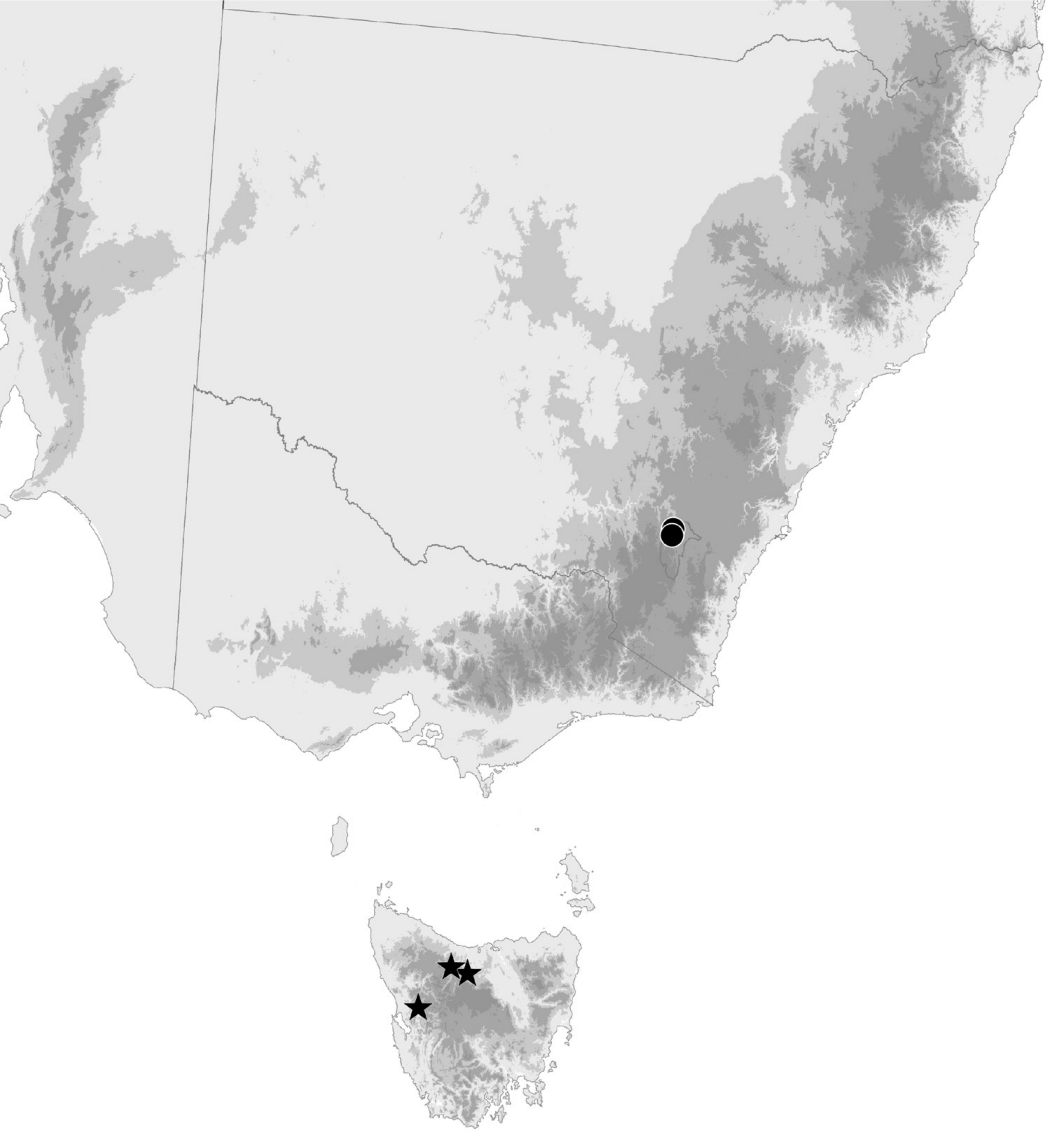
Known geographic distributions of *Tasmanitachoides baehri* and *T. erwini* in southeastern Australia. Circles: *T. baehri*; stars: *T. erwini*.

Figures 10E , 15D , 17B , 18A , 19A , 20A , 21

##### Material examined.

***Holotype*.** Male (ANIC), labeled: “Australia: ACT: Murrumbidgee River, 0.15 km u/s Uriarra Crossing (35°14.717’S, 148°57.135’E 440 m) Washed fr. gravel/under cobbles at river edge. N. Porch, 28 Sep. 2002”, “David R. Maddison DNA5569 DNA Voucher” [pale green paper], “HOLOTYPE Tasmanitachoides baehri Maddison & Porch” [partly handwritten, on red paper]. Genitalia mounted in Euparal on coverslip pinned with specimen; extracted DNA stored separately. GenBank accession numbers for DNA sequences of the holotype are MW291166, MW291260, MW291213, and MW291304.

***Paratypes*** (26). Same label data as holotype (8; ANIC, OSAC). ACT: Murrumbidgee River, 0.15 km u/s Uriarra Crossing (35°14.717'S, 148°57.135'E 440 m). Washed fr. gravel/under cobbles at edge of river. N. Porch, 14 Oct. 2000 (10; NPC, ANIC, ZSM, NMV, NHMUK, MCZ). ACT: Murrumbidgee River, 0.15 km u/s Uriarra Crossing (35°14.717'S, 148°57.135'E 440 m). Washed fr. gravel/under cobbles at edge of river. N. Porch, 28 Sep. 2002 (8; NPC, ANIC, ZSM, NHMUK, MCZ).

##### Other material examined.

We have seen an addition specimen labeled “Paddy’s River, 1 mi. S. of Cotter Dam, ACT, 17.iv.1969. S. Misko” (ANIC; currently in ZSM).

##### Type locality.

Australia: ACT: Murrumbidgee River, 0.15 km u/s Uriarra Crossing (35°14.717'S, 148°57.135'E 440 m).

##### Derivation of specific epithet.

We are honored to name this species after the late Martin Baehr, who discovered and documented many of the carabid species of Australia, and who described 14 of the known species of *Tasmanitachoides*.

##### Diagnosis and description.

Very small, length 1.59–1.63 mm (n = 4). A pale species, body mostly orange, with the front half of the elytra and head a darker reddish orange. Antennae pale testaceous, with antennomeres 5–11 slightly infuscated. Head with moderately long but shallow frontal furrows, reaching approximately the center of the eye, and at least to the anterior supraorbital seta (Fig. 17B); with a groove extending from anterior supraorbital puncture anteriad and mediad to approximately halfway toward the frontal furrow (Fig. 17B). Pronotum convex, narrow, only slightly wider than head (Fig. 10E). Hind angle of pronotum obtuse. Elytra more parallel-sided than *T. wilsoni*. Striae 2 and 3 shallow, broad, impunctate grooves (Figs 15D, 18A); nearby intervals convex. Stria 5 deeply engraved in anterior half of elytron; stria 5 reaching or nearly reaching the second discal seta (ed5; Fig. 15D). Striae 6 and 7 effaced. Discal setae ed6 apparently in stria 2. Microsculpture without engraved lines; where present on the dorsal surface, the microsculpture is formed as low papillae without defined boundaries (Fig. 18A). Pronotum and head very shiny, virtually without microsculpture. Aedeagus (Figs 19A, 20A) with internal sac sclerites compact, and sinuate, very similar to those of *T. wilsoni* (Fig. 19B).

##### Comparison with related species.

Likely to be confused only with similarly small and compact *T. wilsoni*, from which it can be distinguished by the narrower pronotum with less rounded lateral margins, and narrower, less rounded elytra. In addition, *T. wilsoni* has much shorter frontal furrows, which do not reach the anterior supraorbital seta (Fig. 17A); *T. wilsoni* also lacks the notable groove extending forward from the anterior supraorbital seta. The elytral striae in *T. wilsoni* are less evident than in *T. baehri*: *T. baehri* has an evident (if shallow and broad) stria 3 between the two anterior discal setae (Figs 15D, 18A), whereas in *T. wilsoni* it is either absent or extremely faint and shallow (Fig. 15C); stria 5 in *T. wilsoni* is much shorter, only reaching to around half-way in between the two anterior discal setae (Fig. 15C), as opposed to reaching or nearly reaching the second discal seta (ed5) as it does in *T. baehri* (Fig. 15D) *T. baehri* and *T. wilsoni* look very much like small members of the tribe Tachyini (e.g., *Elaphropus*, *Tachyura*). The two *Tasmanitachoides* can be distinguished by the presence of four setae on the clypeus, as opposed to the two setae present in tachyines.

##### Geographic distribution.

Only known from the Australian Capital Territory (Fig. 21), but very likely occurring in similar habitats in NSW.

##### Habitat.

Collected from pockets of gravelly cobble at the edge of still water of the Murrumbidgee River. The collection locality was amongst riverbank sheoaks (*Allocasuarina*) and relatively protected. Specimens were recovered by splashing the gravel bank after removal of cobbles. The species was collected with *T. murrumbidgensis*, *T. rufescens*, and a single specimen of *T. leai*.

##### Phylogenetic relationships.

This species belongs to the *kingi* species group and appears to be sister to *T. wilsoni* among the sampled species (Figs 5–9).

##### Notes.

This species was called “Tasmanitachoides cf. rufescens” in Maddison et al. (2019).

#### Tasmanitachoides erwini sp. nov.

Taxon classificationAnimaliaColeopteraCarabidae

BC7D7FFF-74C2-5896-A285-A134C22A8F60

http://zoobank.org/5FF236BF-1E87-4480-8798-230E71470E66

Figures 1 , 2C , 12C , 16B , 17G , 18B , 19C , 20B, C , 21

##### Material examined.

***Holotype*.** Male (ANIC), labeled: “Australia: Tasmania: River Forth at C136, 41.4712°S 146.1366°E, 126 m, 14.i.2019. DRM 19.012. D.R. Maddison & N.A. Porch”, “David R. Maddison DNA5509 DNA Voucher” [pale green paper], “HOLOTYPE Tasmanitachoides erwini Maddison & Porch” [partly handwritten, on red paper]. Genitalia mounted in Euparal on coverslip pinned with specimen; extracted DNA stored separately. GenBank accession numbers for DNA sequences of the holotype are MW291170, MW291262, MW291215, MW291234, and MW291305.

***Paratypes*** (23). Same label data as holotype (20; ANIC, OSAC, NPC, ZSM, NMV, QVMAG, NHMUK, TMAG, USNM). In addition to these, we have seen three additional specimens, all in the MCZ, which we have designated as paratypes. Two are a labeled “L. StClaire-Queenstown Jan. ’57 Tas Darlingtons” “Tachyshobarti (Sl.) det Darl. ‘61”; according to Darlington (1962:117) these two specimens are from the crossing of the King River by the Queenstown road, which at the time (before the Crotty Dam) would have been approximately 42.074°S 145.652°E. The third is labeled “Mersey R Vy. Mar. ’57 Tas Darlingtons” “Tachyshobarti (Sl.) det Darl. ‘61”. According to the map in Darlington (1960), this locality is at approximately 41.532°S, 146.426°E. These specimens formed Darlington’s concept of *Tasmanitachoides hobarti*. They also are specimens studied and figured by Erwin (1972) as *T. hobarti*.

##### Type locality.

Australia: Tasmania: at the mouth of Machinery Creek into the River Forth at road C136, 41.4712°S, 146.1366°E, 126 m.

##### Derivation of specific epithet.

We are honored to name this species after the late Terry Lee Erwin, for his many contributions to carabidology and systematics in general, and to our knowledge of *Tasmanitachoides* and other bembidarenines in particular.

##### Diagnosis and description.

Length 2.25–2.75 mm (n = 7); most specimens less than 2.6 mm. One of the darker species of *Tasmanitachoides* (Fig. 1): body piceous to black; appendages piceous, including basal antennomeres, with the exception of the tarsi, which are slightly paler. Body relatively flat and parallel-sided; elytra narrowing posteriorly, and thus more pointed than other species. Head without tubercles at anterior corners of clypeus, and without concave region in anterior half. Frontal furrows (Fig. 17G) more or less straight, reaching backward to approximately the center of the eye, parallel or slightly diverging posteriorly; bottom of furrows rugose. Pronotum relatively narrow (Fig. 12C), slightly sinuate laterally in front of the right or slightly acute hind angle. First stria abruptly sinuate, very close to the suture in the anterior fifth or fourth, at which point it abruptly bends away from the suture. Striae 3 and 4 very weak, almost absent in some specimens; the striae 3 and 4 are joined at the anterior discal seta (ed3; Fig. 16B), and in most specimens are merged in front of that point. Stria 5 distinctly engraved throughout the entire anterior half; in posterior half it gradually weakens toward the rear. Stria 6 consisting of a few isolated punctures; stria 7 absent. Discal setae ed6 in stria 3. Microsculpture weak, sculpticells weakly engraved, and thus the surface is shiny; sculpticells isodiametric on head and pronotum, slightly longitudinally stretched on elytra (Fig. 18B). Aedeagus (Figs 19C, 20B, C) with internal sac sclerites elongated and relatively straight, very similar to those of *T.* sp. “Lerderderg R” (Fig. 19D). Ventral surface of the aedeagus quite straight (Fig. 20B, C).

##### Comparison with related species.

As with other members of the *wattsensis* group, this species has a relatively unmodified clypeus, without anterior lateral tubercles, and with the third and fourth elytral striae nearly effaced. Its darker color (including the entirely piceous antenna) and flatness distinguish it from other members of the group. It is the only known species of the group from Tasmania. From the other two large and dark *Tasmanitachoides* from Tasmania, *T. hobarti* and *T. leai*, *T. erwini* is distinguished by having a darker antennomere 1 and flatter body. From *T. hobarti* it is further distinguished by the much weaker striae 3 and 4; from *T. leai* by the longer stria 5.

##### Geographic distribution.

Only known from northwestern Tasmania (Fig. 21).

##### Habitat.

At the type locality, members of this species were found during daylight hours in fine gravel on the banks of Mineral Creek at its mouth into the River Forth (Fig. 2C); specimens were found after splashing the gravel with water. The banks had no visible vegetation. Present in the same habitat were *Tasmanitachoides leai*, *T. kingi*, and *T.* sp. “River Forth”.

##### Phylogenetic relationships.

This species belongs to the *wattsensis* species group, and appears to be the sister to *T.* sp. “Lerderderg R” among the sampled species (Figs 5–9).

##### Notes.

This is the species illustrated by Erwin (1972) as *T. hobarti*. This is evident both by the localities of the specimens he examined (as the localities match Darlington’s), and because of the figures themselves, including the features of the genitalia, which match those of this species rather than *T. hobarti*. The male genitalia of “*Tasmanitachoides hobarti*” figured in Baehr (1990: Fig. 12) is of this species as well. Some specimens from the type series (collected 14 January 2019) are teneral.

#### *Tasmanitachoides angulicollis* Baehr *and T. hendrichi* Baehr

We have examined the holotype of *T. hendrichi* and a paratype of *T. angulicollis*, and found them to be extremely similar; it is possible that they are synonyms.

#### *Tasmanitachoides comes* Baehr and *T. gerdi* Baehr

There is only one known specimen of *T. comes* and only one of *T. gerdi* (Baehr 2010). In Martin Baehr’s collection in ZSM, one of those specimens (Fig. 11C) exactly matches the description of *T. comes*, including in the pattern of punctures of the head (compare Fig. 11C to Baehr 2010: fig. 4). However, the locality label on the pin with that specimen matches that listed in Baehr (2010) as the type locality of *T. gerdi* (Mt. Elliot, QLD), and the pin bears a label declaring it to be the holotype of *T. gerdi*. In contrast, the specimen matching the description and figures of *T. gerdi* (Fig. 11D) bears the locality label (Little Panton R., WA) and holotype label of *T. comes*. The simplest explanation is that the labels were accidentally switched at some point. In resolving whatever accidents of history yielded the contradiction between description and labels, the published description, including figures, take precedence, and as there is no doubt to which specimens Baehr was referring in his 2010 description, the specimen in our Fig. 11C should be considered the holotype of *T. comes*, and the specimen in our Fig. 11D should be considered the holotype of *T. gerdi*. The specimens we are calling T. cf. gerdi may be *T. gerdi*, but we await more detailed study of the holotype, and better understanding of the distribution of *Tasmanitachoides* species, before we can be more definitive.

#### *Tasmanitachoides hobarti* (Blackburn) and *T. glabellus* Baehr

We have examined a photograph of the type (or syntype – see Baehr 1990) of *Bembidium hobarti* Blackburn in the NHMUK (courtesy of Beulah Garner), and from that photograph it is clear that the type is conspecific with the large, convex species we are treating here as *T. hobarti*. However, specimens collected by Philip Darlington in Tasmania, and illustrated by Erwin (1972), are *T. erwini*, not *T. hobarti*, and Baehr’s (1990) concept of *T. hobarti* included *T. erwini*, as noted above. It is not clear if Baehr’s concept of *T. hobarti* included true *T. hobarti*. Before our fieldwork in 2019, the only specimens of true *T. hobarti* in museums of which we are aware are members of the type series (NHMUK), and the only specimens of *T. erwini* those in the MCZ. Based upon a search by DRM in 2019, Baehr’s collection (ZSM) includes neither *T. hobarti* nor *T. erwini*, although there is a specimen of *T. kingi* from the Meander River, Tasmania, identified by Baehr in 2011 as *T. hobarti*. Baehr thus apparently had a mixed concept of “*Tasmanitachoides hobarti*” which may or may not have included true *T. hobarti*.

Baehr’s mixed concept of *T. hobarti* may be relevant to understand the history of *T. glabellus*. We have examined high-quality photographs of the paratype of *T. glabellus* (in ZSM, courtesy of Michael Balke), and it looks extremely similar, if not identical, to true *T. hobarti*. We could see no evident differences. As it seems very unlikely that a species would be known from only Tasmania and one mountain top in North Queensland, even given how poorly *Tasmanitachoides* is collected, it seems more likely that these are distinct species or that the label data for the two *T. glabellus* specimens is in error. We leave it to further field work and closer examination of the types to resolve the status of *T. glabellus*.

#### *Tasmanitachoides leai* (Sloane) and *T. hackeri* Baehr

In his description of *T. hackeri*, Baehr (2008) notes that this species has “Stria 5 near base deeply sulcate, abruptly ended behind basal third”, and indeed, the paratype from the type locality that we have examined (ZSM) has this trait. This is the character by which he separates this species from, for example, *T. leai* in the dichotomous key he presents. However, in this regard *T. hackeri* exactly matches all specimens of *T. leai* we have examined, including the lectotype (ANIC), as *T. leai* also has a short, abruptly ending stria 5, against Baehr’s (2008) and later keys. We can find no significant differences between the paratype of *T. hackeri* and *T. leai*, and it is likely that they are synonyms. However, we do not formally synonymize them now, awaiting study of additional specimens from NSW and QLD.

#### *Tasmanitachoides maior* Baehr

The only specimen Baehr (1990) had seen of *T. maior* was a female 2.9 mm in length. We have measured seven additional specimens, and found that Baehr’s female is at the upper end of the range; the specimens we measured range from 2.44 to 2.89 mm in length.

#### *Tasmanitachoides wattsensis* (Blackburn) and relatives

The specimens we have in hand of the *T. wattsensis* group from Victoria and New South Wales show a great deal of variation, hinting at a complex of closely related species. The specimens from the Lerderderg River (*T.* sp. “Lerderderg R”) are distinctly broader than the remainder, and appear to be a separate species. This is the species that was called “Tasmanitachoides cf. leai” in Maddison et al. (2019). East and north of Melbourne are other forms, including true *T. wattsensis* (of which the specimen shown in Fig. 12A may be a member). Additional research will be necessary to understand the diversity in this complex.

## Concluding remarks

George Eugene Ball died on 12 January 2019, as the two authors of this current paper were travelling on the ferry from Melbourne, Victoria to Devonport, Tasmania, in the midst of the field work that produced the bulk of the specimens on which this paper was based. In less than two years since that day, the world has lost most of the remaining senior figures in carabid systematics, and in the process a tremendous amount of knowledge about carabid beetles that had never been written down. George’s death was followed by that of Martin Baehr, who knew the Australian carabid fauna better than anyone. We lost Augusto Vigna Taglianti and Ross Taylor Bell later in 2019. In the early spring of 2020 we lost Terry Lee Erwin, and in early October, Shun-Ichi Uéno. To have lost six of our grand masters in less than two years is stunning. Our naming a species after Terry and one after Martin are but small gestures to help us honor and remember these two carabidologists, and all the others, like George, Augusto, Ross, and Shun-Ichi, who have devoted their lives to uncover the hidden diversity in the small organisms with which we share our planet.

## Supplementary Material

XML Treatment for Tasmanitachoides baehri

XML Treatment for Tasmanitachoides erwini

## Supplementary materials

Supplementary material 1 Mesquite NEXUS file containing the DNA sequence data and resulting phylogenetic trees from maximum likelihood analyses for *Tasmanitachoides* and other Bembidarenini Data type NEXUS file (DNA sequences, phylogenetic trees) https://binary.pensoft.net/file/555144This dataset is made available under the Open Database License (http://opendatacommons.org/licenses/odbl/1.0/). The Open Database License (ODbL) is a license agreement intended to allow users to freely share, modify, and use this Dataset while maintaining this same freedom for others, provided that the original source and author(s) are credited.David R. Maddison, Nick Porch

## References

[B1] Baehr M (1990) Revision of the Australian ground-beetle genus *Tasmanitachoides* Erwin (Insecta: Coleoptera: Carabidae: Bembidiinae), with special regard to the tropical species. Invertebrate Taxonomy 4: 867–894. 10.1071/IT9900867

[B2] Baehr M (2001) *Tasmanitachoides* Erwin *glabellus* n. sp. from north Queensland, Australia, with a note on *Tasmanitachoides lutus* (Darlington) (Insecta, Coleoptera, Carabidae, Bembidiinae). Animal Biodiversity and Conservation 24: 1–7. https://www.raco.cat/index.php/ABC/article/download/57568/67536/

[B3] Baehr M (2008a) A new species of the tachyine genus *Tasmanitachoides* Erwin from northern New South Wales, Australia (Coleoptera, Carabidae, Bembidiinae, Tachyini). Mitteilungen der Münchner Entomologischen Gesellschaft 98: 121–126. https://www.zobodat.at/pdf/MittMuenchEntGes_098_0121-0126.pdf

[B4] Baehr M (2008b) Two new species of the genus *Tasmanitachoides* Erwin from north Queensland, Australia (Insecta: Coleoptera: Carabidae: Bembidiini). Annals of Carnegie Museum 77: 13–19. 10.2992/0097-4463-77.1.13

[B5] Baehr M (2009) A new species of tachyine genus *Tasmanitachoides* from the Kimberley Division, Western Australia (Coleoptera, Carabidae). Records of the Western Australian Museum 25: 159–164. 10.18195/issn.0312-3162.25(2).2009.159-164

[B6] Baehr M (2010) Two new species of the genus *Tasmanitachoides* Erwin, 1972 from northern Australia (Coleoptera, Carabidae, Trechitae). Entomologische Blaetter fuer Biologie und Systematik der Kaefer 106: 25–39.

[B7] Baehr M (2013) New species and new records of the genus *Tasmanitachoides* Erwin from Australia (Insecta, Coleoptera, Carabidae, Trechitae). Mitteilungen der Münchner Entomologischen Gesellschaft 103: 85–94. https://www.zobodat.at/pdf/MittMuenchEntGes_103_0085-0094.pdf

[B8] Blackburn T (1901) Further notes on Australian Coleoptera, with descriptions of new genera and species. XXIX. Transactions of the Royal Society of South Australia 25: 99–131. https://www.biodiversitylibrary.org/page/16140789#page/113/mode/1up

[B9] Chakrabarty P Warren M Page LM Baldwin CC (2013) GenSeq: An updated nomenclature and ranking for genetic sequences from type and non-type sources. ZooKeys: 29–41. 10.3897/zookeys.346.5753 PMC3821064 24223486

[B10] Darlington Jr PJ (1960) Australian carabid beetles IV. List of localities, 1956–1958. Psyche 67: 111–126. 10.1155/1960/81583

[B11] Darlington Jr PJ (1962) Australian carabid beetles XI. some *Tachys*. Psyche 69: 117–128. 10.1155/1962/71454

[B12] Erwin TL (1972) Two new genera of bembidiine carabid beetles from Australia and South America with notes on their phylogenetic and zoogeographic significance (Coleoptera). Breviora 383: 1–19. https://www.biodiversitylibrary.org/page/3664881#page/87/mode/1up

[B13] Grebennikov VV (2008) *Tasmanitachoides* belongs to Trechini (Coleoptera: Carabidae): discovery of the larva, its phylogenetic implications and revised key to Trechitae genera. Invertebrate Systematics 22: 479–488. https://pdfs.semanticscholar.org/e36f/0d032fdc88ad3e0b0f78ec9f5409eccabe6d.pdf

[B14] Green P (1999) Phrap. Version 0.990329. http://phrap.org

[B15] Green P Ewing B (2002) Phred. Version 0.020425c. http://phrap.org.

[B16] Kalyaanamoorthy S Minh BQ Wong TKF von Haeseler A Jermiin LS (2017) ModelFinder: fast model selection for accurate phylogenetic estimates. Nature Methods 14: 587–589. 10.1038/nmeth.4285 28481363 PMC5453245

[B17] Kanda K Pflug JM Sproul JS Dasenko MA Maddison DR (2015) Successful recovery of nuclear protein-coding genes from small insects in museums using Illumina sequencing. PLoS ONE 10: e0143929. 10.1371/journal.pone.0143929 PMC4696846 26716693

[B18] Katoh K Standley DM (2013) MAFFT Multiple Sequence Alignment Software Version 7: Improvements in Performance and Usability. Molecular Biology and Evolution 30: 772–780. 10.1093/molbev/mst010 23329690 PMC3603318

[B19] Maddison DR (1993) Systematics of the Holarctic beetle subgenus Bracteon and related *Bembidion* (Coleoptera: Carabidae). Bulletin of the Museum of Comparative Zoology 153: 143–299. https://www.biodiversitylibrary.org/page/4280989#page/157/mode/1up

[B20] Maddison DR (2008) Systematics of the North American beetle subgenus Pseudoperyphus (Coleoptera: Carabidae: *Bembidion*) based upon morphological, chromosomal, and molecular data. Annals of Carnegie Museum 77: 147–193. 10.2992/0097-4463-77.1.147

[B21] Maddison DR (2012) Phylogeny of *Bembidion* and related ground beetles (Coleoptera: Carabidae: Trechinae: Bembidiini: Bembidiina). Molecular Phylogenetics and Evolution 63: 533–576. 10.1016/j.ympev.2012.01.015 22421212

[B22] Maddison DR Anderson R (2016) Hidden species within the genus *Ocys* Stephens: the widespread species *O. harpaloides* (Audinet-Serville) and *O. tachysoides* (Antoine) (Coleoptera, Carabidae, Bembidiini). Deutsche Entomologische Zeitschrift 63: 287–301. 10.3897/dez.63.10748

[B23] Maddison DR Cooper KW (2014) Species delimitation in the ground beetle subgenus Liocosmius (Coleoptera: Carabidae: *Bembidion*), including standard and next-generation sequencing of museum specimens. Zoological Journal of the Linnean Society 172: 741–770. 10.1111/zoj.12188

[B24] Maddison DR Kanda K Boyd OF Faille A Porch N Erwin TL Roig-Juñent S (2019) Phylogeny of the beetle supertribe Trechitae (Coleoptera: Carabidae): Unexpected clades, isolated lineages, and morphological convergence. Molecular Phylogenetics and Evolution 132: 151–176. 10.1016/j.ympev.2018.11.006 30468941

[B25] Maddison DR Maddison WP (2020a) Chromaseq: a Mesquite package for analyzing sequence chromatograms. Version 1.52. http://chromaseq.mesquiteproject.org

[B26] Maddison DR Maddison WP (2020b) Zephyr: a Mesquite package for interacting with external phylogeny inference programs. Version 3.11. http://zephyr.mesquiteproject.org

[B27] Maddison DR Ober KA (2011) Phylogeny of minute carabid beetles and their relatives based upon DNA sequence data (Coleoptera, Carabidae, Trechitae). ZooKeys 147: 229–260. 10.3897/zookeys.147.1871 PMC3286250 22379388

[B28] Maddison DR Sproul JS (2020) Species delimitation, classical taxonomy and genome skimming: a review of the ground beetle genus *Lionepha* (Coleoptera: Carabidae). Zoological Journal of the Linnean Society 189: 1313–1358. 10.1093/zoolinnean/zlz167

[B29] Maddison WP Maddison DR (2020c) Mesquite: a modular system for evolutionary analysis. Version 3.61. http://mesquiteproject.org

[B30] Moulton JK Wiegmann BM (2004) Evolution and phylogenetic utility of CAD (rudimentary) among Mesozoic-aged Eremoneuran Diptera (Insecta). Molecular Phylogenetics and Evolution 31: 363–378. 10.1016/S1055-7903(03)00284-7 15019631

[B31] Nguyen L-T Schmidt HA von Haeseler A Minh BQ (2015) IQ-TREE: A fast and effective stochastic algorithm for estimating maximum-likelihood phylogenies. Molecular Biology and Evolution 32: 268–274. 10.1093/molbev/msu300 25371430 PMC4271533

[B32] Shull VL Vogler AP Baker MD Maddison DR Hammond PM (2001) Sequence alignment of 18S ribosomal RNA and the basal relationships of Adephagan beetles: Evidence for monophyly of aquatic families and the placement of Trachypachidae. Systematic Biology 50: 945–969. 10.1080/106351501753462894 12116642

[B33] Sloane TG (1895) Studies in Australian entomology No. VII. New Genera and species of Carabidae (Including some notes on previously described species, and synoptic lists of genera and species). Proceedings of the Linnean Society of New South Wales 9: 393–455. 10.5962/bhl.part.18118

[B34] Sloane TG (1896) On the Australian Bembidiides referable to the genus *Tachys*, with the description of a new allied genus *Pyrrotachys*. Proceedings of the Linnean Society of New South Wales 21: 355–377. 10.5962/bhl.part.8477

[B35] Sloane TG (1921) Revisional notes on Australian Carabidae. Part VI. Tribe Bembidiini. Proceedings of the Linnean Society of New South Wales 46: 192–208. 10.5962/bhl.part.14008

[B36] Smith MA Bertrand C Crosby K Eveleigh ES Fernandez-Triana J Fisher BL Gibbs J Hajibabaei M Hallwachs W Hind K Hrcek J Huang D-W Janda M Janzen DH Li Y Miller SE Packer L Quicke D Ratnasingham S Rodriguez J Rougerie R Shaw MR Sheffield C Stahlhut JK Steinke D Whitfield J Wood M Zhou X (2012) *Wolbachia* and DNA barcoding insects: patterns, potential, and problems. PLoS ONE 7: e36514. 10.1371/journal.pone.0036514 PMC3342236 22567162

[B37] Talavera G Castresana J (2007) Improvement of phylogenies after removing divergent and ambiguously aligned blocks from protein sequence alignments. Systematic Biology 56: 564–577. 10.1080/10635150701472164 17654362

[B38] Thalmann O Hebler J Poinar HN Pääbo S Vigilant L (2004) Unreliable mtDNA data due to nuclear insertions: a cautionary tale from analysis of humans and other great apes. Molecular Ecology 13: 321–335. 10.1046/j.1365-294X.2003.02070.x 14717890

